# Comparative transcriptomic analysis revealed potential mechanisms regulating the hypertrophy of goose pectoral muscles

**DOI:** 10.1016/j.psj.2024.104498

**Published:** 2024-11-02

**Authors:** Xinyue Hu, Yali Liu, Bincheng Tang, Jiwei Hu, Hua He, Hehe Liu, Liang Li, Shenqiang Hu, Jiwen Wang

**Affiliations:** State Key Laboratory of Swine and Poultry Breeding Industry, College of Animal Science and Technology, Sichuan Agricultural University, Chengdu, Sichuan, PR China

**Keywords:** Pectoral muscle, Muscle hypertrophy, Goose, Transcriptome

## Abstract

Pectoral muscle development is an important economic trait. According to the different essence, muscle development can be divided into 2 processes: embryonic muscle fiber generation and postnatal muscle fiber hypertrophy, and postnatal muscle fiber hypertrophy has a greater impact on muscle development than the number of muscle fibers formed during the embryonic phase in poultry. However, the underlying mechanisms regulating the hypertrophy of goose pectoral muscles have not been elucidated. Therefore, the purpose of the present study was to conduct transcriptome sequencing in pectoral muscles of both Landes (LD) and Sichuan White (SW) geese at 6, 10, and 30 weeks of age to reveal the molecular mechanisms regulating pectoral muscle hypertrophy through intra-breed and inter-breed bioinformatics analyses. Phenotypically, the pectoral muscle weight/index of LD and SW geese increased from 6 to 30 weeks of age, and except for the pectoral muscle index at 10 weeks of age (*P* = 0.962), at the same age, the pectoral muscle weight/index of LD geese were significantly higher than that of SW geese (*P* < 0.05). In transcriptional regulation, intra-breed bioinformatics analysis identified 3331 genes whose expression levels were opposite to the trend of pectoral muscle hypertrophy both in LD and SW geese, and the 3331 genes were mainly enriched into abundant KEGG pathways related to lipid metabolism, proliferation/apoptosis, and immune response. Moreover, 23 genes (including *SLC2A10, TNFRSF1A, PRKAA1, SLC27A4, ITGB2, THY1, RHOA, MYL10, ACTB, PRKCB, PIK3R2, RAC2, DMD, LATS2, YAP1, WWTR1, SMAD7, CTGF, FGF1, AXIN2, GLI2, ID2*, and *CCND2*) who were enriched in 6 crosstalk pathways named viral myocarditis, insulin resistance, sphingolipid signaling pathway, hippo signaling pathway, chemokine signaling pathway, and leukocyte transendothelial migration were identified as the key candidate genes regulating the hypertrophy of goose pectoral muscles. In inter-breed bioinformatics analysis, abundant different expression genes (DEGs) related to lipid metabolism, immune response, and proliferation/apoptosis were identified between LD and SW geese too, and compared with SW geese, the expression level of *MYL10* in LD geese was lower, while the expression levels of *GLI2*/*CTGF*/*SMAD7* in LD geese were higher. These results suggested that the hypertrophy of goose pectoral muscles might be achieved through more lipid deposition and less leukocyte infiltration to promote the proliferation of cells within the muscles, and the low expression of *MYL10* and high expressions of *GLI2*/*CTGF*/*SMAD7* might the keys to induce the pectoral muscle hypertrophy of LD geese from 6 to 30 weeks of age over that of SW geese. All data the present study obtained will provide new insights into the molecular mechanisms regulating the hypertrophy of goose pectoral muscles.

## Introduction

Meat production is the main purpose of livestock farming, and poultry meat is increasingly popular with consumers because it is affordable and free from religious and cultural restrictions (Petracci and Cavani, 2012). With the market demand continues to increase in global, more and more pressures are being put on breeders and farmers to increase the growth rate and muscle production of poultry (Cavani, et al., 2009; Petracci and Cavani, 2012). Over the past few decades, extensive breeding works had been carried out on poultry, which caused chickens and turkeys marketed in about half the time, as well as double the weight (Barbut, et al., 2008). Noticeably, compared with chickens and turkeys, goose meat is often seen as a “functional food” because it contains more unsaturated fatty acids and minerals that contribute to human health (Geldenhuys, et al., 2013; Goluch and Haraf, 2023; Petracci and Cavani, 2012). However, although goose meat has excellent nutritional value, due to its late muscle development characteristic (Lilja, 1981), the price of goose meat is relatively high, so it is not as popular as other poultry meats (Wereńska, et al., 2022).

According to the different essence, muscle development can be divided into 2 stages: the generation of muscle fibers during the embryonic phase and the hypertrophy of muscle fibers after birth (Zhang, et al., 2014). In the first stage, myocytes who are formed by myoblasts derived from somites through a series of migration, proliferation, and differentiation are able to fuse with each other to form myotubes with multiple cell nuclei (Chal and Pourquié, 2017; Dedieu, et al., 2002), and each myotube will further differentiate into a muscle fiber (Buckingham, 2017; Chal and Pourquié, 2017). Previous study has shown that the number of muscle fibers in animals rarely increase after birth, which suggested that the proliferation and differentiation of myoblasts during embryonic phase would affect muscle development (Gu, et al., 2024). Therefore, in recent years, breeders have mainly explored embryos and revealed a large number of mechanisms regulating the proliferation and differentiation of myoblasts in geese (Chen, et al., 2022; Guo, et al., 2021; Huang, et al., 2024; Wang, et al., 2022; Xu, et al., 2021). While as for muscle fiber hypertrophy, Smith (1963) pointed out that the number and size of muscle fibers in large-sized chickens were higher than those in small-sized chickens at 10 weeks of age, and the effect of muscle fiber size on muscle weight was superior to the number of muscle fibers, which hinted that revealing the regulatory mechanisms of postnatal muscle fiber hypertrophy may be more helpful to understand the development of pectoral muscles in geese.

Worldwide, geese can be divided into European geese originated from the Greylag Goose (*Anser anser*) and Chinese geese originated from the Swan Goose (*Anser cygnoides*) (Ottenburghs, et al., 2016). Both European and Chinese geese have formed many varieties with different physical appearance, reproductive efficiency, and growth rate during the long-term domestication process (Deng, et al., 2021). Among them, Landes geese (LD, one variety of European geese with faster growth rate) and Sichuan White geese (SW, one variety of Chinese geese with slower growth rate) are often used for commercial breeding due to their excellent production performances. Therefore, the aim of the present study was to construct the mRNA expression profiles of pectoral muscles in LD and SW geese at the young stage (6 weeks of age), the marketed stage (10 weeks of age), and the body maturation stage (30 weeks of age) by using transcriptome sequencing technology, and to reveal the molecular mechanisms regulating the hypertrophy of goose pectoral muscles through intra-breed and inter-breed bioinformatics analyses.

## Materials and methods

### Ethics statement

All experimental procedures including animal handling were approved by the Institutional Animal Care and Use Committee (IACUC) of Sichuan Agricultural University (Chengdu Campus, Sichuan, China) with Approval No. DKY20170913.

### Management of experimental geese

In the present study, 60 SW/LD male geese that were reared in the Waterfowl Breeding Experimental Farm of Sichuan Agricultural University (Ya'an, Sichuan, China) were selected as experimental materials. The 120 geese were hatched in the same batch, and after they emerged from shell, they were firstly raised in brooder houses at a constant temperature of 31 °C until 2 weeks of age. Then, all geese were moved to an indoor area (length × width: 6 × 13 m) consisting of a 60 m² cement playground and an 18 m² fermentation bed to raise until 30 weeks of age. These geese had free access to food and water throughout the feeding process, and the nutritional composition information of diets at different stages was shown in Table 1.

**Table 1:** Nutritional composition of basal diets.

Items	Stage (0 - 21 days of age)	Stage (21 - 210 days of age)
H_2_O ( %)	14.00	14.00
Crude protein ( %)	19.00	16.00
Crude fiber ( %)	6.00	8.00
Crude ash ( %)	8.00	11.00
Total Phosphorus ( %)	0.50	0.40
Total Calcium ( %)	0.70 - 1.50	0.70 - 1.50
NaCl ( %)	0.30 - 0.80	0.30 - 0.80
Methionine ( %)	0.42	0.36

### Sample collection

Respectively, at 6, 10, and 30 weeks of age, 8 SW/LD male healthy geese were randomly selected to slaughter after their body weight were determined. At slaughter, geese were firstly anesthetized with carbon dioxide, and then were sacrificed by carotid artery bloodletting. After geese were sacrificed, the left and right pectoral muscles were separated to weight immediately, and the pectoral muscle index was calculated according to the following formula: pectoral muscle index = bilateral pectoral muscle weight (g)/body weight (g) × 100 %. In addition, 1 g tissue from the same location of the left pectoral muscle in each goose was collected to store at -80 °C after rapidly freezing it in liquid nitrogen.

### Total RNA extraction, library preparation, and sequencing

According to the manufacturer's instructions, total RNAs were extracted from 18 (3 SW/LD male geese among 6, 10, and 30 weeks of age) pectoral muscle samples by using RNeasy Mini Kit (Qiagen, Beijing, China), and NanoDrop 2000 Microultraviolet Spectrophotometer (Thermo Fisher Scientific, Wilmington, USA) was utilized to detect RNA concentration; Agilent 2100 Bioanalyzer (Agilent Technologies, Santa Clara, USA) was used to detect RNA integrity. Then, 20 ng of total RNAs were extracted from each sample to construct sequencing libraries by going through the following processes in turn: 1) The ribosomal RNAs were removed by using Epicenter RiboZeroTM rRNA Removal Kit (Epicenter, WI, USA); 2) NEBNextR UltraTM Directional RNA Library Prep Kit (New England Biolabs, MA, USA) was used to construct cDNA libraries; 3) AMPure XP system (Beckman Coulter, MA, USA) was used to selectively reserve cDNA fragments between 150 bp and 200 bp length as sequencing libraries. Finally, paired-end sequencing of libraries was performed on an Hiseq 2500 platform (Illumina, Sandiego, USA) to gain raw reads.

### Quantitative reverse transcription-PCR (RT-qPCR) validation

In order to prove the reliability and repeatability of sequencing data, 7 candidate genes (including *ACTB, CTGF, MYL10, RHOA, SMAD7, WWTR1*, and *SLC27A4*) that were identified as closely related to goose pectoral muscle hypertrophy were selected for RT-qPCR verification. The HiScript® III RT Kit (Vazyme, Nanjing, China) was used to convert mRNA into cDNA. Primer 5.0 software was used to design primers (Supplementary Table S1) spanning exon. Expression levels of the 7 genes were detected by SYBR Green method in the Bio-Rad CFX96 real-time PCR system (Bio-Rad, Hercules, USA). The *GAPDH* was used as housekeeping gene and 3 technical replicates were performed. Systems of qPCR reaction were as follows: SYBR Green PCR SuperMix (Vazyme, Nanjing, China) was 10 µL, both PCR forward and reverse primers (10 µM) were 0.4 µL, ddH2O was 7.2 µL, cDNA was 2 µL. The 2^-△△CT^ method was used to analyze RT-qPCR data (Pfaffl, 2001).

### Bioinformatics analysis

FastQC software (version 0.11.9) was used to filter reads containing adapter/ploy-N and low quality in raw reads to obtain clean reads (Kim, et al., 2018). HISAT2 (version 2.2.1) software was used to map clean reads to the reference genome of Sichuan White goose (data being published) to obtain sequencing alignment/mapping (SAM) files (Kim, et al., 2015). SAMtools (version 1.6.0) software was used to convert SAM files into binary alignment/mapping (BAM) files (Li, et al., 2009). FeaturCounts (version 1.6.0) software was utilized to gain the count of each transcript, and GenomicFeatures (version 1.46.3) package was used to normalize the count of each transcript as transcripts per million (TPM) (Liao, et al., 2014). Principal component analysis (PCA) was performed based on the Vegan (version 2.6.4) package to compare differences between samples (Ringnér, 2008). STEM (version 1.3.11) software was used to perform time series analysis of mRNA expression profiles (Ernst and Bar-Joseph, 2006). DESeq2 (1.34.0 version) package was used to identify the different expression genes (DEGs) in pectoral muscles between SW and LD geese at the same age, and the screening criteria were |log2(Fold change)| > 1 and *P* value < 0.05 (Love, et al., 2014). Functional enrichment analysis of Kyoto Encyclopedia of Genes and Genomes (KEGG) was performed by using KOBAS (version 3.0) online software (Mao, et al., 2005). The KEGG and STRING 11.5 databases were used to determine the relationships of KEGG pathways and genes (Szklarczyk, et al., 2023), respectively. Cytoscape (version 3.10.1) software was used to visualize the protein-protein interaction (PPI) networks (De Marinis, et al., 2022).

### Statistical analysis

Statistical analysis was performed by using SPSS 27.0 (IBM, Chicago, USA) software. The body weight and pectoral muscle weight/index of SW and LD geese among 6, 10, and 30 weeks of age were subjected to ANOVA testing, and the significances of body weight and pectoral muscle weight/index between SW and LD geese at the same age were analyzed by t-test. Graph Pad Prism (version 8.0) software was used to plot pictures and the results in pictures were expressed as mean ± standard deviation. In addition, *P* < 0.05 was considered a statistically significant difference in the present study.

## Results

### The regularity of pectoral muscle hypertrophy in LD and SW geese

**Fig 1:**
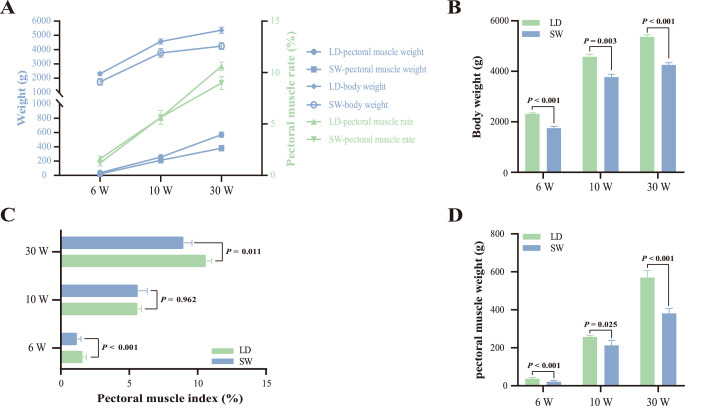
Anatomical results of pectoral muscles in SW and LD geese at 6, 10, and 30 weeks of age. (A) Changes of pectoral muscle weight/index in SW and LD geese from 6 to 30 weeks of age. (B) Difference of pectoral muscle weight between SW and LD geese at 6, 10, or 30 weeks of age. (C) Difference of pectoral muscle index between SW and LD geese at 6, 10, or 30 weeks of age. Abbreviations: LD, Landes goose; SW, Sichuan White goose; W, weeks of age.

Anatomical results showed that the body weight and pectoral muscle weight/index of SW and LD geese exhibited a same change regularity from 6 to 30 weeks of age: all were increasing (Fig 1A). Moreover, at the same age, the body weight and pectoral muscle weight/index of LD geese were significantly higher than that of SW geese (*P* < 0.05, Fig 1B-D), except for the pectoral muscle index at 10 weeks of age (*P* = 0.962, Fig 1C).

### Overview of RNA-seq data

As shown in Table 2, 527,978,189 raw reads were obtained from 18 pectoral muscle samples by transcriptome sequencing, and 334,728,816 clean reads were retained after quality control. Respectively, the Q20 (percentage of reads with Phred quality value > 20), Q30 (percentage of reads with Phred quality value >30), GC content, and mapping rate of the 18 samples ranged from 97.73 % to 98.37 %, 92.46 % to 94.21 %, 48.13 % to 52.18 %, and 88.57 % to 93.90 %. In addition, PCA results showed that the mRNA expression patterns in pectoral muscles of geese existed age and breed differences (Supplementary Fig S1).

**Table 2:** Basic information of RNA-seq data.

Sample^1^	Raw reads	Clean reads	Q20 ( %)	Q30 ( %)	GC content ( %)	Mapping rate ( %)
LD-6W-1	28,212,762	17,759,983	97.85	92.61	50.61	90.05
LD-6W-2	49,935,104	27,977,904	98.04	93.22	50.16	89.16
LD-6W-3	25,422,098	19,266,606	98.02	93.18	48.47	91.28
LD-10W-1	25,402,581	21,104,358	97.83	92.66	48.13	92.51
LD-10W-2	35,035,065	18,170,308	98.00	93.16	50.17	90.83
LD-10W-3	30,344,609	16,586,444	98.14	93.50	50.27	89.80
LD-30W-1	39,368,390	24,246,097	98.05	93.15	51.22	88.57
LD-30W-2	27,647,046	22,226,103	97.73	92.46	49.46	90.75
LD-30W-3	27,692,714	15,140,791	98.09	93.38	51.13	89.22
SW-6W-1	23,836,633	12,785,081	97.90	92.65	52.18	89.25
SW-6W-2	19,335,756	10,710,615	98.37	94.21	49.70	91.51
SW-6W-3	26,264,559	20,094,346	98.05	93.29	48.56	91.98
SW-10W-1	22,694,470	18,451,112	97.98	93.05	48.90	93.24
SW-10W-2	28,296,573	15,985,220	98.08	93.36	49.36	93.90
SW-10W-3	30,228,219	15,984,358	98.17	93.60	50.72	91.22
SW-30W-1	28,217,083	22,786,596	98.01	93.19	49.30	92.91
SW-30W-2	32,128,142	19,199,282	98.18	93.54	50.03	91.64
SW-30W-3	27,916,385	16,253,612	98.27	93.91	50.49	92.00

1 Abbreviations: LD, Landes goose; SW, Sichuan White goose; W, weeks of age.

### Identification of genes regulating the pectoral muscle hypertrophy of geese

**Fig 2:**
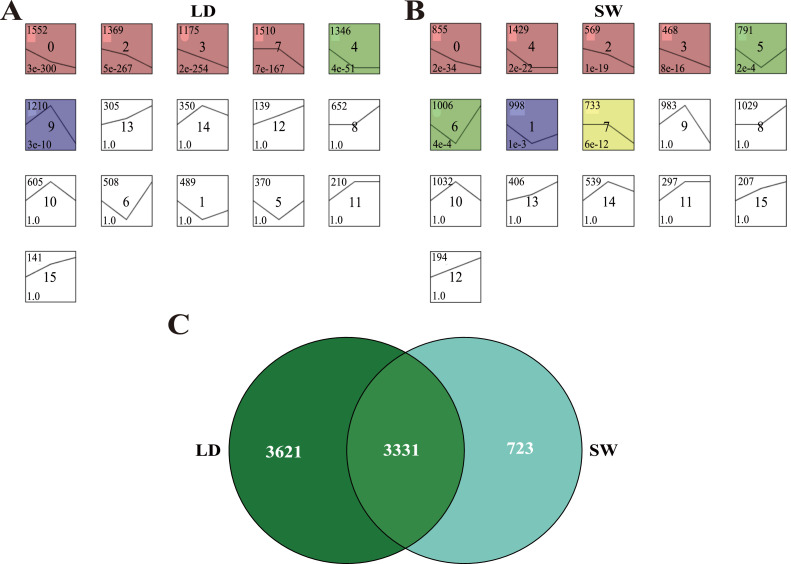
Time series analysis of pectoral muscle mRNA expression profiles. The trend distribution of mRNA expression level in pectoral muscles of LD (A) and SW (B) geese from 6 to 30 weeks of age. (C) Venn diagram of genes that were classified into common/significant trends in LD and SW geese. Abbreviations: LD, Landes goose; SW, Sichuan White goose.

The time series analysis of pectoral muscle mRNA expression profiles in LD and SW geese both obtained 16 trend maps (Fig 2A-B). Among them, 0, 2, 3, 4, 7, and 9 trends were significantly enriched in LD geese (*P* < 0.05, Fig 2A), while 0, 1, 2, 3, 4, 5, 6, and 7 trends were significantly enriched in SW geese (*P* < 0.05, Fig 2B). The 0, 2, 3, 4, and 7 trends were significantly enriched in both LD and SW geese, and the gene expression levels in these 5 trends decreased from 6 to 30 weeks of age (Fig 2A-B), which was contrary to the actual hypertrophy trend of pectoral muscles. Subsequently, further analysis showed that 3331 genes were shared among the 5 trends in LD and SW geese (Fig 2C).

### Functional analysis of genes regulating the pectoral muscle hypertrophy of geese

As illustrated in Supplementary Table S2, the 3331 genes were significantly enriched to 176 KEGG pathways (*P* < 0.05). Among them, 30 pathways including AGE-RAGE signaling pathway in diabetic complications, glycosaminoglycan biosynthesis-chondroitin sulfate/dermatan sulfate, N-Glycan biosynthesis, phospholipase D signaling pathway, choline metabolism in cancer, etc. were associated with lipid metabolism; 47 pathways such as pathways in cancer, MAPK signaling pathway, TGF-beta signaling pathway, hippo signaling pathway, and Rap1 signaling pathway were associated with proliferation/apoptosis; 47 pathways for example focal adhesion, human papillomavirus infection, human T-cell leukemia virus 1 infection, Fc gamma R-mediated phagocytosis, and adherens junction were associated with immune response.

### Network analysis of genes regulating the pectoral muscle hypertrophy of geese

**Fig 3:**
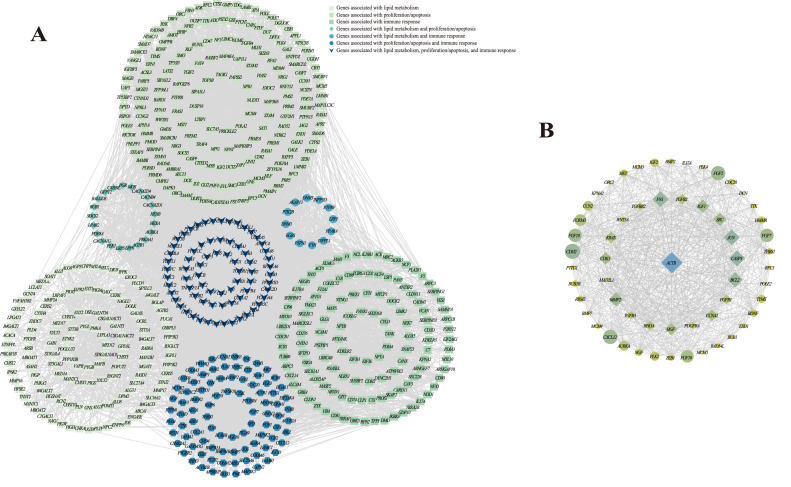
Network analysis of genes that were enriched into KEGG pathways associated with lipid metabolism, proliferation/apoptosis, and immune response. (A) PPI networks of all genes that were enriched into KEGG pathways associated with lipid metabolism, proliferation/apoptosis, and immune response. (B) The most pivotal PPI subnetwork identified based on MCC algorithm. The larger the size of shape, the more degree the protein encoded by corresponding gene.

**Fig 4:**
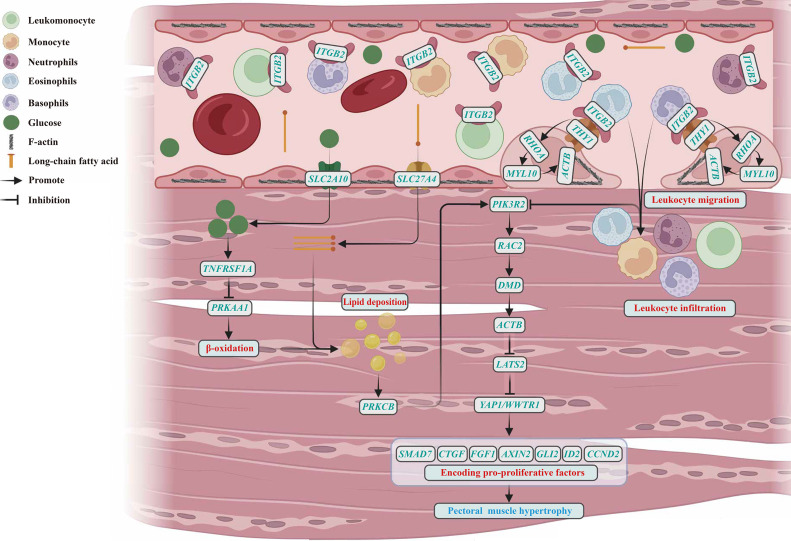
Regulation network involved in the hypertrophy of goose pectoral muscles.

PPI network analysis was used to explore interactions of genes (a total of 667 genes) associated with lipid metabolism, proliferation/apoptosis, or immune response. As shown in Fig 3A, this PPI network was consisted of 646 nodes and 6699 edges, which could be further divided into 21 subnetworks by the MCODE plug-in (Table 3). Among them, the subnetwork with the highest score was composed of 58 nodes and 800 edges, and the degree of protein encoded by *ACTB* was the highest (Fig 3B). In order to clarify the molecular network regulating the hypertrophy of goose pectoral muscles, researchers observed the distributions of *ACTB* in the previously enriched KEGG pathways, and found that *ACTB* seemed to regulate muscle hypertrophy mainly through the viral myocarditis pathway. From the KEGG database, it can be seen that there was crosstalk in viral myocarditis, insulin resistance, sphingolipid signaling pathway, hippo signaling pathway, chemokine signaling pathway, and leukocyte transendothelial migration pathways. Therefore, 23 genes (including *SLC2A10, TNFRSF1A, PRKAA1, SLC27A4, ITGB2, THY1, RHOA, MYL10, ACTB, PRKCB, PIK3R2, RAC2, DMD, LATS2, YAP1, WWTR1, SMAD7, CTGF, FGF1, AXIN2, GLI2, ID2*, and *CCND2*) enriched in the 6 pathways were selected to construct an interaction network diagram (Fig 4).

**Table 3:** Basic information of PPI subnetworks.

Subnetwork	Score	Nodes	Edges	Genes
1	28.07	58	800	*NGF, AURKA, PTTG1, FGF10, FOXM1, CCN2, WNT5A, TTK, DCN, IGF1, CASP3, POLE2, CCNA2, ACTB, BUB1, MCM5, PLK1, RHOA, CXCL12, MCM4, MAD2L1, CDK1, IGF2, PLK4, CDC20, THBS1, FGFR1, EXO1, PDGFRA, BDNF, BUB1B, CDH2, KPNA2, MCM3, BMP2, FGF2, TGFB2, JUN, RFC3, RAD54L, FGF18, ZEB1, TGFB3, MMP2, RRM2, BMP7, KRAS, ORC1, MET, IL17A, TGFBR2, FN1, HMMR, FGF7, SRC, TYMS, BCL2, HGF*
2	9.64	12	53	*CHST14, CHPF, DSE, B4GALT7, EXT2, CSGALNACT2, CHST3, TNXB, CSGALNACT1, XYLT1, CHSY1, XYLT2*
3	8.61	47	198	*FZD7, LTBP1, VIM, ITGA9, SMURF2, COL6A2, PIK3R2, ITGA11, RYK, IGFBP3, COL6A6, ABL1, SOCS3, COL2A1, BGLAP, TNFRSF1A, THBS3, SERPINF1, SMURF1, GAB1, FZD4, FBN1, COL6A3, AXIN2, BMP6, DVL3, EGR1, TERT, ILK, VCAN, COL6A1, LMNB1, COL4A5, LAMC1, PTCH1, LRP6, WNT5B, FGFR4, SMO, FMOD, LAMB1, ITGAV, FZD1, BMPR1B, LEF1, BAMBI, COL4A6*
4	6.53	20	62	*ADK, PDE1A, DCK, DMD, NT5C3B, ITGB2, PDE8A, CTSS, SPI1, TLN1, CD47, TIAM1, PDE7A, RAC2, APBB1IP, APRT, PDE10A, CD2, ENTPD1, TLN2*
5	6.35	30	92	*POLE3, BMPR1A, COL3A1, DBF4, PDGFA, THY1, BMPR2, SMC3, BUB3, TGFBR1, SGO1, ACVR2A, RPA2, FGF1, PIK3R1, POLE, SMAD6, RFC5, COL1A2, RFC2, POLA2, NRG1, CALML4, CSF1R, ACVR2B, PLCG1, LAMA2, NTRK2, CSF1, ACVR1*
6	6.00	6	15	*TANK, IKBKE, CYLD, TRAF1, TBK1, MAVS*
7	4.77	48	112	*MBOAT1, ALG1, NAGLU, CACNA2D1, CERS5, ARSB, GMPPB, PLD1, SERPING1, UGT8, LPAR2, CERS2, GNS, SPTLC1, EFNA5, TBXA2R, MBOAT2, GALC, LCLAT1, AGTR2, GALNS, PYGB, GALT, CERK, TFPI, ASAH2, GNE, S1PR2, PYGL, PIGH, PLD4, HGSNAT, LAMP2, GRK5, IDS, LPAR1, PIGW, F5, SELENOI, GFPT2, CACNB4, PIGG, CACNA2D4, UAP1, ALG8, SGPL1, PTGFR, PIGP*
8	4.11	38	76	*SOCS2, RASA1, GNG12, YES1, GNA11, LY96, RNASEL,* SEC61A1*, CARD11, STING1, VAV2, CD81, XCR1, PLCD3, PI4KA, TRAF3, PDIA4, PDIA3, NCF4, CD74, INPP5D, CISH, FYB1, SYNJ2, RUNX1, P2RY12, INPPL1, CADM1, NF1, PTPN6, SEC13, NOD1, FYN, SIGLEC1, OCRL, SMARCB1, NLRX1, CASP2*
9	4.00	4	6	*IPPK, PPIP5K2, IP6K2, PPIP5K1*
10	4.00	4	6	*FREM1, FREM2, FRAS1, NPNT*
11	3.75	9	15	*DOLK, MGAT1, ALG11, STT3B, STT3A, PIGM, UGDH, GALE, DPM2*
12	3.63	28	49	*TSC2, MYD88, CX3CR1, AKT3, RAD54B, PPARA, PTPN1, PPP2R5D, YAP1, AGTR1, CD80, PPP2R5C, TNFSF10, PTK2B, MAPK8, SMAD7, GSN, TCF7L2, PMS2, ACE, RAD52, RICTOR, FST, CTSK, INHBA, ITGB3, THBS2, NCAM1*
13	3.33	7	10	*CAD, GMPS, PDE9A, DPYD, NPR2, CTPS2, NPR1*
14	3.33	28	45	*ALG5, POFUT1, B4GALT1, RBPJ, A4GALT, HDAC2, PHF10, ARPC1B, MAN1B1, MYO10, SMARCE1, TSTA3, ABCA1, NCOR2, ARPC3, ALG6, EXOC7, NCSTN, FCSK, MED14, B3GNT2, ARPC5, EXOC2, C1GALT1, EXOC1, GMDS, MED17, SMARCD2*
15	3.00	3	3	*MLH1, BARD1, XPA*
16	3.00	3	3	*CMPK1, DGUOK, DCTD*
17	3.00	3	3	*ATP6V1C2, TCIRG1, ATP6V0E1*
18	3.00	3	3	*ARHGEF7, GIT2, STEAP3*
19	3.00	3	3	*F3, SERPIND1, PROS1*
20	3.00	3	3	*FRMD6, AMOT, LATS2*
21	3.00	3	3	*CTSZ, HEXA, NAGA*

### Molecular mechanism causing pectoral muscle hypertrophy of LD geese over that of SW geese

**Fig 5:**
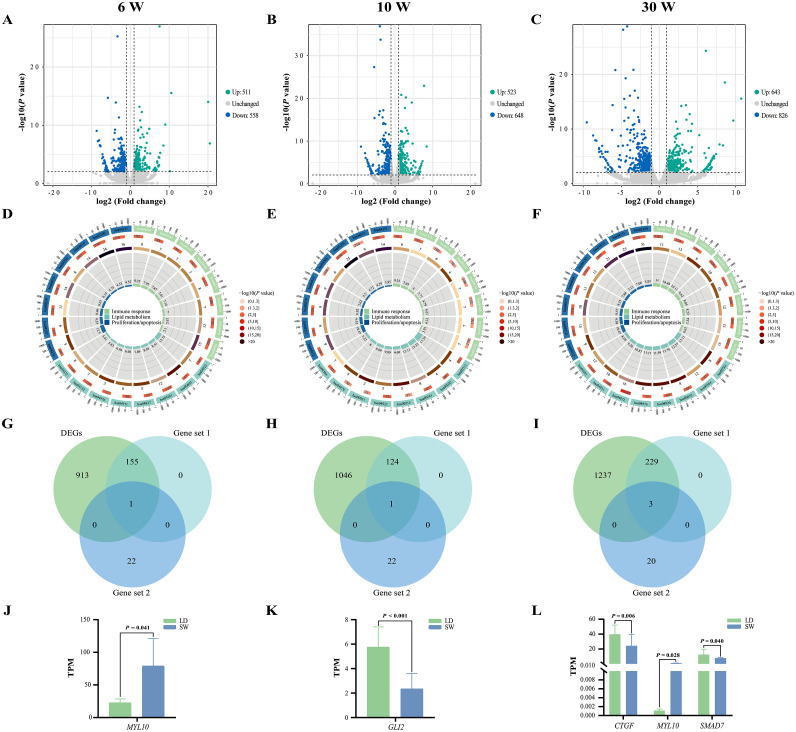
Bioinformatics analysis of pectoral muscle mRNA expression profiles between SW and LD geese at same age. Volcano diagram of DEGs that were screened from 6 (A), 10 (B), or 30 (C) weeks of age. Some KEGG pathways enriched by DEGs that were identified from 6 (D), 10 (E), or 30 (F) weeks of age were involved in lipid metabolism, proliferation/apoptosis, and immune response. Venn diagram of DEGs, DEGs that were enriched into KEGG pathways associated with lipid metabolism, proliferation/apoptosis, and immune response, and candidate genes that were involved in the hypertrophy of goose pectoral muscles at 6 (G), 10 (H), or 30 (I) weeks of age. (J) Difference of *MYL10* expression level between SW and LD geese at 6 weeks of age. (K) Difference of *GLI2* expression level between SW and LD geese at 10 weeks of age. (L) Difference of *CTGF, MYL10,* and *SMAD7* expression levels between SW and LD geese at 30 weeks of age. Data was displayed as “mean ± standard deviation” in figures J-L. Abbreviations: W, weeks of age; DEGs, different expression genes; LD, Landes goose; SW, Sichuan White goose; TPM, transcripts per million. “Gene set 1″ represents DEGs that were enriched into KEGG pathways associated with lipid metabolism, proliferation/apoptosis, and immune response; “Gene set 2″ represents candidate genes that were involved in the hypertrophy of goose pectoral muscles.

In the present study, 1069, 1171, and 1469 DEGs were identified from the pectoral muscles between SW and LD geese at 6, 10, and 30 weeks of age (Fig 5A-C), respectively. Subsequently, functional enrichment analysis showed that the DEGs identified from all 3 ages were significantly enriched into a large number of KEGG pathways related to immune response, lipid metabolism, and proliferation/apoptosis (*P* < 0.05, Fig 5D-F, Supplementary Table S3). In addition, venn diagram showed that the expression level of *MYL10* involved in the interaction network diagram was significantly different between SW and LD geese (Fig 5G), and its expression level in LD geese was significantly lower than that in SW geese at 6 weeks of age (*P* < 0.05, Fig 5J); the expression level of *GLI2* involved in the interaction network diagram was significantly different between SW and LD geese (Fig 5H), and its expression level in LD geese was significantly higher than that in SW geese at 10 weeks of age (*P* < 0.05, Fig 5K); the expression levels of *MYL10, CTGF*, and *SMAD7* involved in the interaction network diagram were significantly different between SW and LD geese (Fig 5I), and the expression level of *MYL10* in LD geese was significantly lower than that in SW geese (*P* < 0.05, Fig 5L), while the expression levels of *CTGF* and *SMAD7* in LD geese were significantly higher than those in SW geese at 30 weeks of age (*P* < 0.05, Fig 5L). Moreover, the expression levels of the 7 key candidate genes that generated from RT-qPCR were similar to the RNA-seq results (Supplementary Figure S2), indicating the reliability of RNA-seq results.

## Discussion

Phenotypically, the pectoral muscle weight/index of SW and LD geese increased from 6 to 30 weeks of age, indicating that pectoral muscles were ever-hypertrophying until geese reaching the body maturation, which was consistent with the results obtained in Beijing-You chickens (Li, et al., 2020), Jingyuan chickens (Yu, et al., 2024), and Peking ducks (Zhang, et al., 2014). In addition, no matter at 6 weeks of age, 10 weeks of age, or 30 weeks of age, the pectoral muscle weight of LD geese was always significantly higher than that of SW geese, this result was consistent with a report pointed that pectoral muscle development of LD geese was superior to that of SW geese after treatment with min-maximum normalization method (Yan, et al., 2023).

As the main raw material of poultry meat products, muscle development determines the quality and quantity of poultry meat products for human consumption (Huang, et al., 2024; Wang, et al., 2021), which has caused a large number of studies aimed at resolving the underlying mechanisms regulating muscle fiber formation that can affect the development of muscles in poultry (Cao, et al., 2023; Guo, et al., 2021; Tan, et al., 2024). Nevertheless, compared with the number of muscle fibers formed in embryonic phase, postnatal muscle fiber hypertrophy has more influences on muscle development (Smith, 1963). In the present study, 3331 genes whose expression levels were opposite to the trend of pectoral muscle hypertrophy were identified from the pectoral muscle mRNA expression profiles of LD and SW geese at 6, 10, and 30 weeks of age. Functional analysis showed that the 3331 genes were mainly enriched into a large number of KEGG pathways related to lipid metabolism, proliferation/apoptosis, and immune response, suggesting that the hypertrophy of goose pectoral muscles might be mainly affected by the lipid metabolism, proliferation/apoptosis, and immune response of body. Furtherly, PPI analysis revealed functional interactions between genes enriched in the above pathways related to lipid metabolism, proliferation/apoptosis, and immune response, and the gene named *ACTB* was identified as a hub gene regulating the hypertrophy of goose pectoral muscles, which was consistent with previous findings that the knockout of *ACTB* could reduce the expression level of dystrophin protein to cause amyotrophy (Prins, et al., 2011; Rice and McNair, 2010). Moreover, in order to reveal the molecular network of *ACTB* regulating the hypertrophy of goose pectoral muscles, the viral myocarditis was identified as a pivotal pathway because it reflects the direct link between *ACTB* and *DMD* who can encode dystrophin protein. Subsequently, combined with KEGG database and known reports, it was found that there was crosstalk between the viral myocarditis and 5 pathways, including insulin resistance (Luo, et al., 2019), sphingolipid signaling pathway (Tan-Chen, et al., 2020), hippo signaling pathway (Watt, et al., 2018), chemokine signaling pathway (De Paepe and De Bleecker, 2013), and leukocyte transendothelial migration (Li and Luo, 2019), which had been confirmed to be involved in regulating muscle development. These results showed that the 6 pathways should be the key to mediating the hypertrophy of goose pectoral muscles.

When the present study attempted to explain how the 6 pathways affected the hypertrophy of goose pectoral muscles, researchers noticed that 23 genes (including *SLC2A10, TNFRSF1A, PRKAA1, SLC27A4, ITGB2, THY1, RHOA, MYL10, ACTB, PRKCB, PIK3R2, RAC2, DMD, LATS2, YAP1, WWTR1, SMAD7, CTGF, FGF1, AXIN2, GLI2, ID2*, and *CCND2*) have mutual regulating relationships. As shown in Fig 4: blood vessels that transport materials such as glucose, fatty acid, and leukocyte (a group of cells composed of neutrophils, eosinophils, basophils, monocyte, and leukomonocyte) are widely distributed between the muscle fibers of pectoral muscles in poultry (Hadad, et al., 2014; Pugsley and Tabrizchi, 2000). Among them, glucose could be transported to muscle fibers via the glucose transporter 10 encoded by *SLC2A10* and promote the expression level of *TNFRSF1A* (also known as *TNFR*) (Gao, et al., 2020; McMillin, et al., 2017), while the TNFα treatment targeting *TNFR* could reduce the protein expression level encoded by *PRKAA1* (also known as *AMPKα1*) in skeletal muscle cells to inhibit β-oxidation of fatty acids, thereby affecting lipid deposition in muscle fibers (Steinberg, et al., 2006). Long-chain fatty acid could be transported to muscle fibers via fatty acid transport protein 4 encoded by *SLC27A4*, which could directly promote lipid deposition in muscle fibers (Benninghoff, et al., 2020). In addition, integrin encoded by *ITGB2* in the cell membrane of leukocytes could bind specifically to integrin receptor protein encoded by *THY1* in the cell membrane of vascular endothelial cells (Fekadu, et al., 2022; Wetzel, et al., 2004). The combination of integrin and its receptor would assist the contraction of F-actin encoded by *ACTB* through stimulating the expression level of *MYL10* after activating RHOA signal in endothelial cells (Izawa, et al., 2018), thereby improving vascular permeability to promote the leukocytes infiltrating into muscle fibers (Heemskerk, et al., 2016). Notably, both lipid deposition and leukocyte infiltration were able to affect the expression level of *PIK3R2*: lipid deposition could indirectly enhance *PIK3R2* expression through *PRKCB* (also known as *PKCβII*) (Jaishy, et al., 2015; Zhang, et al., 2023), while leukocyte infiltration could directly inhibit *PIK3R2* expression (Liu, et al., 2022). It has been reported that *PIK3R2* could positively regulate the expression level of *RAC2* (Boccarelli, et al., 2024), and the expression level of *DMD* would increase when *RAC2* with high expression level (Oakley, et al., 2019). Campbell (2008) pointed out that the dystrophin protein encoded by *DMD* could assist the F-actin encoded by *ACTB* to connect to extracellular matrix and hinder muscle necrosis. Besides, F-actin was epistatic to *LATS2* (Wada, et al., 2011), F-actin could inhibit the phosphorylation of YAP1/WWTR1 proteins by *LATS2* and increase the activity of YAP1/WWTR1 proteins to stimulate the expression level of pro-proliferative genes including *SMAD7* (Cohen, et al., 2015), *CTGF* (Wang, et al., 2019), *FGF1* (Liu, et al., 2024), *AXIN2* (Nie, et al., 2019), *GLI2* (Burton, et al., 2024), *ID2* (Matsumura, et al., 2002), and *CCND2* (Sun, et al., 2023; Watt, et al., 2018). Previous report also pointed out that the hypertrophy of muscles in poultry after birth depended on the proliferation of cells within the muscles, especially satellite cells (Velleman, et al., 2010). These results suggested that the hypertrophy of pectoral muscles in postnatal geese might be mainly achieved through more lipid deposition and less leukocyte infiltration to promote the proliferation of cells within the muscles. Interestingly, no matter at 6 weeks of age, 10 weeks of age, or 30 weeks of age, numerous DEGs related to lipid metabolism, immune response, and proliferation/apoptosis were identified from the pectoral muscles between LD geese with larger pectoral muscles and SW geese with smaller pectoral muscles, and the expression level of *MYL10* in LD geese was lower than that in SW geese at 6 weeks of age; the expression level of *GLI2* in LD geese was higher than that in SW geese at 10 weeks of age; the expression level of *MYL10* in LD geese was lower than that in SW geese, while the expression levels of *CTGF* and *SMAD7* were higher than that in SW geese at 30 weeks of age. These results not only provided further support for above speculation, but also suggested that compared with SW geese, the rapid hypertrophy of pectoral muscles in LD geese might be caused by the low expression level of *MYL10*, which promoted the proliferation of cells within the muscles through reducing the leukocyte infiltration in pectoral muscles.

In conclusion, the present study found that pectoral muscles of both LD and SW geese hypertrophied continuously from 6 to 30 weeks of age, and the hypertrophy of pectoral muscles in LD geese was better than this in SW geese. In addition, intra-breed bioinformatics analysis showed that 23 genes (including *SLC2A10, TNFRSF1A, PRKAA1, SLC27A4, ITGB2, THY1, RHOA, MYL10, ACTB, PRKCB, PIK3R2, RAC2, DMD, LATS2, YAP1, WWTR1, SMAD7, CTGF, FGF1, AXIN2, GLI2, ID2*, and *CCND2*) identified from viral myocarditis, insulin resistance, sphingolipid signaling pathway, hippo signaling pathway, chemokine signaling pathway, and leukocyte transendothelial migration pathways were pivotal for the hypertrophy of goose pectoral muscles, and the 23 genes might promote the proliferation of cells within the muscles by increasing lipid deposition and reducing leukocyte infiltration, thereby regulating the hypertrophy of goose pectoral muscles. Meanwhile, inter-breed bioinformatics analysis indicated that compared with SW geese, the rapid hypertrophy of pectoral muscles in LD geese might be caused by the low expression of *MYL10* and high expressions of *GLI2*/*CTGF*/*SMAD7*. These results would help better understand the underlying molecular mechanisms regulating the hypertrophy of the goose pectoral muscles.

## Funding

This research was supported by China Agriculture Research System of MOF and MARA (CARS-42-4) and Key Technology Support Program of Sichuan Province (2021YFYZ0014).

## Declaration of competing interest

The authors declare that the research was conducted in the absence of any commercial or financial relationships that could be construed as a potential conflict of interest.

## Appendix Supplementary materials

Image, application 1

Image, application 2

Image, application 3

Image, application 4

Image, application 5
