# Supplementary material for: Comparative transcriptomic analysis revealed potential mechanisms regulating the hypertrophy of goose pectoral muscles
Source: Poult Sci. 2024 Nov 2;103(12):104498. doi: 10.1016/j.psj.2024.104498 (PMC11577216; doi:10.1016/j.psj.2024.104498)
Supplement: Supplementary file 1 [file mmc1.docx]

***Supplementary Figure S1. Principal component analysis of pectoral muscle mRNA expression profiles.*** (A) Principal component analysis of pectoral muscle mRNA expression profiles in SW and LD geese at 6,10, and 30 weeks of age. (B) Principal component analysis of pectoral muscle mRNA expression profiles between SW and LD geese at 6 weeks of age. (C) Principal component analysis of pectoral muscle mRNA expression profiles between SW and LD geese at 10 weeks of age. (D) Principal component analysis of pectoral muscle mRNA expression profiles between SW and LD geese at 30 weeks of age. Abbreviations: LD, Landes goose; SW, Sichuan White goose; W, weeks of age.
