# Supplementary material for: Comparative transcriptomic analysis revealed potential mechanisms regulating the hypertrophy of goose pectoral muscles
Source: Poult Sci. 2024 Nov 2;103(12):104498. doi: 10.1016/j.psj.2024.104498 (PMC11577216; doi:10.1016/j.psj.2024.104498)
Supplement: Supplementary file 2 [file mmc2.docx]

***Supplementary Figure S2. Expression levels of ACTB, CTGF, MYL10, RHOA, SMAD7, WWTR1, and SLC27A4 that were detected by RT-qPCR and RNA-seq.*** Data were displayed as “mean ± standard deviation” in figures. Abbreviations: LD, Landes goose; SW, Sichuan White goose; W, weeks of age.
