# Supplementary material for: Comparative transcriptomic analysis revealed potential mechanisms regulating the hypertrophy of goose pectoral muscles
Source: Poult Sci. 2024 Nov 2;103(12):104498. doi: 10.1016/j.psj.2024.104498 (PMC11577216; doi:10.1016/j.psj.2024.104498)
Supplement: Supplementary file 3 [file mmc3.docx]

***Supplementary Table S1. Primers used in this study.***

| Primer name | Sequence (5′ - 3′) | Product length (bp) |
| --- | --- | --- |
| *MYL10*-F | GAACAGGGACGGCTTTATTG | 269 |
| *MYL10*-R | CTGCCTGTGTCATAAGCATTTC |  |
| *CTGF*-F | AACCAGGGTCACCAACGATA | 273 |
| *CTGF*-R | TCAGGGCACTTGAACTCCAC |  |
| *ACTB*-F | GCGTGACATCAAGGAGAAGC | 190 |
| *ACTB*-R | GCAGGACTCCATACCCAAGA |  |
| *RHOA*-F | GTGATGGTGCCTGTGGAAAG | 157 |
| *RHOA*-R | TCTTGTCCTGCTGTATCCCAC |  |
| *SMAD7*-F | TCACCAAGGAAGTGGACGG | 132 |
| *SMAD7*-R | TGATGGAAAAACCTGGAAACAC |  |
| *WWTR1*-F | CACCAGCACCAAATAACACC | 165 |
| *WWTR1*-R | TCTAATTCGCTCCCTCTCCA |  |
| *SLC27A4*-F | ATGGAGTCCCGCAATCAGTA | 222 |
| *SLC27A4*-R | TCCTTCCCCTGACCAGTAGA |  |
| *GAPDH*-F | CATGTTCGTGATGGGTGTG | 239 |
| *GAPDH*-R | CTGGGATAATGTTCTGGGC |  |
