# Supplementary material for: Comparative transcriptomic analysis revealed potential mechanisms regulating the hypertrophy of goose pectoral muscles
Source: Poult Sci. 2024 Nov 2;103(12):104498. doi: 10.1016/j.psj.2024.104498 (PMC11577216; doi:10.1016/j.psj.2024.104498)
Supplement: Supplementary file 4 [file mmc4.docx]

***Supplementary Table S2. Functional enrichment analysis of genes whose expression levels were contrary to the actual hypertrophy trend of pectoral muscles both in LD and SW geese.***

| KEGG pathway | Rich factor | *P* value | Genes |
| --- | --- | --- | --- |
| **Lipid metabolism** | | | |
| AGE-RAGE signaling pathway in diabetic complications | 0.230 | 2.94E-06 | *TGFB2, TGFB3, PLCG1, AKT3, COL3A1, EGR1, JUN, PIK3R2, PIK3R1, MAPK8, TGFBR2, TGFBR1, PRKCB, COL4A6, COL4A5, PLCD3, KRAS, CASP3, FN1, COL1A2, AGTR1, MMP2, BCL2* |
| Glycosaminoglycan biosynthesis-chondroitin sulfate/dermatan sulfate | 0.500 | 1.01E-05 | *CHPF, CHST14, CHSY1, XYLT2, XYLT1, CHST3, DSE, CSGALNACT2, B4GALT7, CSGALNACT1* |
| N-Glycan biosynthesis | 0.300 | 1.11E-05 | *DOLK, ALG8, ALG1, ALG6, ALG5, MAN1B1, MGAT1, MAN1C1, B4GALT3, STT3B, B4GALT1, ALG11, STT3A, B4GALT2, DPM2* |
| Phospholipase D signaling pathway | 0.182 | 1.83E-05 | *PTGFR, PPAP2B, PDGFRA, PTK2B, AKT3, DNM1, DNM3, TSC2, RHOA, FYN, PIK3R2, PIK3R1, PDGFA, PDGFC, RALGDS, SHC2, MRAS, HGF, PLA2G4A, CYTH4, KRAS, PLD1, ADCY2, PLCG1, LPAR2, AGTR1, LPAR1* |
| Choline metabolism in cancer | 0.212 | 2.24E-05 | *PDGFA, PDGFRA, PDGFC, RAC2, RALGDS, SLC22A3, LYPLA1, MAPK8, JUN, PRKCB, PLCG1, AKT3, PIK3R2, PIK3R1, PPAP2B, HGF, PLA2G4A, KRAS, TSC2, CHKA, PLD1* |
| Glycosaminoglycan degradation | 0.474 | 3.91E-05 | *GNS, ARSB, HEXA, NAGLU, HEXB, GALNS, IDS, HGSNAT, HPSE2* |
| Insulin resistance | 0.194 | 6.77E-05 | *PRKCB, PPP1R3B, RPS6KA6, MGEA5, CREB5, TNFRSF1A, SOCS3, PRKAA1, AKT3, PTPRF, PYGB, CREB3L1, PIK3R2, PIK3R1, PPARA, PTPN1, SLC27A4, GFPT2, PYGL, MAPK8, PPP1CC* |
| Sphingolipid signaling pathway | 0.185 | 8.83E-05 | *AKT3, SGPL1, CERS5, CERS2, ASAH2, S1PR2, BID, FYN, PIK3R2, PIK3R1, SPTLC1, MAPK8, RAC2, PRKCB, RHOA, KRAS, PLD1, TNFRSF1A, PPP2CB, PPP2R5D, PPP2R5C, BCL2* |
| Thyroid hormone signaling pathway | 0.185 | 8.83E-05 | *HDAC1, HDAC2, PLCG1, AKT3, TSC2, MED12L, MED12, MED14, PIK3R2, PIK3R1, MED17, DIO3, SRC, ITGB3, RCN2, PRKCB, PLCD3, KRAS, SLC16A2, ITGAV, PLN, ACTB* |
| Insulin signaling pathway | 0.175 | 9.08E-05 | *PRKAA1, AKT3, TSC2, PPP1R3B, INPPL1, PYGB, PIK3R2, PIK3R1, MAPK8, PYGL, ACACA, SHC2, PHKA2, PRKAR1B, HGF, PTPN1, KRAS, EXOC1, PPP1CC, SOCS3, SOCS2, EXOC7, CALML4, PTPRF* |
| Progesterone-mediated oocyte maturation | 0.182 | 4.43E-04 | *CCNB3, MAD2L1, CCNA2, ADCY2, RPS6KA6, PLK1, IGF1, CDK1, AKT3, PGR, AURKA, PIK3R1, BUB1, MAPK8, MOS, KRAS, ANAPC4, PIK3R2* |
| Other types of O-glycan biosynthesis | 0.364 | 4.45E-04 | *B4GALT2, POGLUT1, POMT1, GXYLT2, B4GALT1, B4GALT3, POFUT1, B3GLCT* |
| Other glycan degradation | 0.389 | 7.36E-04 | *MAN2C1, HEXA, HEXB, HEXDC, FUCA1, NEU2, ENGASE* |
| Glycosylphosphatidylinositol (GPI)-anchor biosynthesis | 0.320 | 8.90E-04 | *PIGG, PIGH, PIGM, GAB1, PIGP, PIGS, PIGW, DPM2* |
| Parathyroid hormone synthesis, secretion and action | 0.170 | 8.97E-04 | *MMP16, PRKCB, LRP6, PLD1, ADCY2, LRP5, MMP17, CREB5, EGR1, BGLAP, CREB3L1, AKAP13, GNA11, MMP24, MAFB, FGFR1, RHOA, BCL2* |
| Glycosaminoglycan biosynthesis-keratan sulfate | 0.429 | 1.19E-03 | *ST3GAL1, B4GALT2, B3GNT2, B4GALT1, B4GALT3, CHST2* |
| Phosphatidylinositol signaling system | 0.162 | 2.63E-03 | *OCRL, INPP5D, PI4KA, INPPL1, PRKCB, CALML4, PPIP5K2, TMEM55A, PLCG1, PPIP5K1, PLCD3, PIK3R1, SYNJ2, IPPK, IP6K2, PIK3R2* |
| Sphingolipid metabolism | 0.213 | 2.99E-03 | *SGPL1, CERS5, CERS2, PPAP2B, ASAH2, UGT8, GALC, CERK, SPTLC1, NEU2* |
| Cushing syndrome | 0.135 | 4.26E-03 | *AGTR1, FZD1, FZD4, AXIN2, RASD1, FZD7, CAMK2D, WNT2, CREB5, GNA11, SCG2, TCF7L1, TCF7L2, CACNA1G, DVL3, CREB3L1, ADCY2, WNT5B, WNT5A, LEF1, PDE8A* |
| Adrenergic signaling in cardiomyocytes | 0.134 | 5.69E-03 | *TNNT2, CALML4, RPS6KA5, ADCY2, CAMK2D, PPP1CC, CREB5, PPP2CB, CACNB4, AKT3, MYL4, CREB3L1, BCL2, PLN, PPP2R5D, AGTR2, AGTR1, CACNA2D1, CACNA2D4, PPP2R5C* |
| Glycosphingolipid biosynthesis-globo and isoglobo series | 0.333 | 7.26E-03 | *HEXA, A4GALT, ST3GAL1, HEXB, NAGA* |
| Ether lipid metabolism | 0.191 | 8.56E-03 | *PLD4, AGPS, PLD1, ENPP6, EPT1, UGT8, PLA2G4A, PAFAH1B2, PPAP2B* |
| Mannose type O-glycan biosynthesis | 0.261 | 8.91E-03 | *CHST10, LARGE, POMT1, B4GALT1, B4GALT3, B4GALT2* |
| Glycerophospholipid metabolism | 0.144 | 1.11E-02 | *PLD4, PLD1, LYPLA1, EPT1, ETNPPL, MBOAT2, MBOAT1, ETNK2, LCLAT1, GPD1L, PLA2G4A, LPGAT1, CHKA, PPAP2B* |
| Cholesterol metabolism | 0.180 | 1.20E-02 | *SOAT1, LRPAP1, VAPB, NPC2, LRP1, PLTP, ABCA1, VDAC3, OSBPL5* |
| AMPK signaling pathway | 0.133 | 1.31E-02 | *CAB39L, RAB8A, CCNA2, CREB5, PPP2CB, IGF1, PRKAA1, LEPR, AKT3, CREB3L1, PIK3R2, PIK3R1, PPP2R5D, ACACA, TSC2, PPP2R5C* |
| Glycosphingolipid biosynthesis-lacto and neolacto series | 0.222 | 1.67E-02 | *B3GNT2, ST3GAL4, B4GALT1, A4GALT, B4GALT3, B4GALT2* |
| Mucin type O-glycan biosynthesis | 0.194 | 2.83E-02 | *GALNT16, GCNT4, ST3GAL1, WBSCR17, C1GALT1, GALNT5* |
| Glycosphingolipid biosynthesis-ganglio series | 0.267 | 2.96E-02 | *HEXA, ST6GALNAC6, ST3GAL1, HEXB* |
| Glycosaminoglycan biosynthesis-heparan sulfate/heparin | 0.208 | 3.46E-02 | *EXT2, XYLT2, EXT1, B4GALT7, XYLT1* |
| **Proliferation/apoptosis** | | | |
| Pathways in cancer | 0.174 | 4.82E-14 | *PMAIP1, BMP2, AKT3, PTGER4, GNG12, GADD45G, KRAS, CAMK2D, FN1, FGFR4, CXCL12, FGFR1, PLD1, ADCY2, CCND2, TCF7L1, TCF7L2, RUNX1, HHIP, PDGFA, RASSF5, MGST1, LAMB1, RAC2, JUN, MST1, PIK3R2, PIK3R1, IGF2, IGF1, COL4A6, COL4A5, FGF7, RHOA, LAMC1, FGF2, FGF1, RPS6KA5, ITGAV, CALML4, HLF, AGTR1, HDAC1, PDGFRA, HDAC2, SMO, PTGER2, ABL1, FZD1, SPI1, FZD4, FZD7, CSF1R, FGF18, PML, MAPK8, FGF10, TGFBR2, LAMA2, RALGDS, APPL1, LEF1, LRP6, LRP5, WNT2, MET, LPAR2, LPAR1, TGFB2, TGFB3, TGFBR1, PLCG1, AXIN2, BID, MLH1, GLI2, DVL3, JAG2, TERT, DAPK3, TRAF1, TRAF3, TRAF4, PRKCB, WNT5B, HGF, WNT5A, CASP3, GNA11, PTCH1, MMP2, BCL2* |
| MAPK signaling pathway | 0.203 | 4.80E-12 | *NGF, TGFB3, STMN1, MAP4K4, TGFBR2, MAP3K6, FGFR1, TGFB2, HGF, DDIT3, GNG12, MAP2K5, BDNF, CACNB4, FGFR4, MAP4K3, DUSP16, MAP3K3, RASA1, JUN, MST1, NTRK2, CACNA1G, FGF18, CACNA2D4, NF1, DUSP7, MAPK8IP3, IRAK4, IGF2, PDGFA, TGFBR1, PDGFC, RAC2, MYD88, TAOK1, CSF1R, RASA2, EFNA5, MAPK8, MRAS, PRKCB, FGF7, PLA2G4A, CACNA2D1, KRAS, FGF2, FGF1, PTPRR, RPS6KA5, CASP3, RPS6KA6, TNFRSF1A, GADD45G, PDGFRA, CSF1, MET, AKT3, IGF1, FGF10* |
| TGF-beta signaling pathway | 0.340 | 1.05E-11 | *TGFB2, TGFB3, ACVR1, TGFBR2, SMAD6, SMAD7, ACVR2B, BMPR1A, BMPR1B, FST, PITX2, SMURF1, SMURF2, ZFYVE16, THBS1, INHBA, NEO1, FMOD, DCN, TGFBR1, LTBP1, FBN1, BAMBI, RHOA, BMP7, BMP6, BMP2, PPP2CB, ID2, BMPR2, TGIF2, ACVR2A* |
| Hippo signaling pathway | 0.266 | 1.08E-11 | *TGFB2, PPP2CB, SMAD7, RASSF6, LATS2, BMPR1B, TGFB3, LEF1, FZD1, DLG3, FZD4, AXIN2, FZD7, PPP1CC, YWHAQ, FRMD1, GLI2, DVL3, TGFBR2, TGFBR1, ITGB2, TP53BP2, FRMD6, ACTB, FGF1, YAP1, BMP7, BMP6, BMP2, BMPR1A, WWTR1, WNT2, CCND2, ID2, TCF7L1, TCF7L2, CTGF, BMPR2, WNT5B, WNT5A, AMOT* |
| Rap1 signaling pathway | 0.229 | 2.04E-11 | *NGF, APBB1IP, PDGFA, CSF1R, RASSF5, PDGFRA, RAPGEF6, AKT3, EVL, THBS1, FGFR4, FYB, TLN2, ITGB2, TLN1, TIAM1, FGF18, MAGI3, PIK3R2, PIK3R1, SIPA1L1, SIPA1L2, CNR1, SRC, ITGB3, PDGFC, RAC2, RALGDS, EFNA5, PRKCB, MRAS, FGF7, RHOA, KRAS, FGF2, FGF1, FGFR1, FGF10, ADCY2, VAV2, CSF1, PLCG1, CALML4, MET, IGF1, LPAR2, ACTB, LPAR1* |
| ECM-receptor interaction | 0.337 | 1.15E-10 | *ITGA9, CHAD, TNXB, ITGB3, ITGA11, LAMB1, COL2A1, THBS2, THBS3, THBS1, RELN, HMMR, LAMA2, FRAS1, CD47, COL4A6, COL4A5, TNC, LAMC1, FN1, ITGAV, NPNT, FREM2, FREM1, COL1A2, COL6A1, COL6A3, COL6A2, COL6A6* |
| PI3K-Akt signaling pathway | 0.175 | 4.56E-10 | *ITGA9, NGF, PPP2CB, CHAD, THBS2, ITGB3, PRKAA1, HGF, ITGA11, PDGFRA, GNG12, COL6A6, LAMB1, BDNF, TSC2, LAMC1, FGF10, YWHAQ, TNXB, THBS3, NTRK2, THBS1, COL2A1, FGF18, RELN, PIK3R1, MYB, CREB5, PIK3R2, IGF2, PDGFA, LAMA2, PDGFC, CSF1R, EFNA5, COL6A1, COL4A5, TNC, FGF7, FGFR4, PHLPP1, KRAS, FGF2, FGF1, FGFR1, LPAR2, LPAR1, FN1, COL4A6, CCND2, CSF1, ITGAV, AKT3, MET, CREB3L1, COL1A2, IGF1, PPP2R5D, BCL2, COL6A3, COL6A2, PPP2R5C* |
| Ras signaling pathway | 0.203 | 1.05E-09 | *NGF, RASSF5, PDGFRA, HGF, PLCG1, AKT3, GNG12, ABL1, BDNF, ABL2, RHOA, RALGDS, TBK1, CSF1R, MST1, NTRK2, RASA1, ETS2, TIAM1, FGF18, RASA2, NF1, MAPK8, FGF10, PIK3R2, IGF2, PDGFA, PDGFC, RAC2, PIK3R1, SHC2, EFNA5, PRKCB, MRAS, FGF7, PLA2G4A, KRAS, FGF2, FGF1, FGFR1, EXOC2, PLD1, CSF1, CALML4, MET, FGFR4, IGF1* |
| Gastric cancer | 0.221 | 5.03E-08 | *TGFB2, TGFB3, HGF, AKT3, FZD1, FZD4, AXIN2, FZD7, MLH1, GADD45G, DVL3, FGF18, PIK3R2, PIK3R1, FGF10, TGFBR2, TGFBR1, SHC2, FGF7, LEF1, FGF2, FGF1, KRAS, TERT, LRP6, LRP5, WNT2, TCF7L1, TCF7L2, MET, WNT5B, WNT5A, BCL2* |
| Hepatocellular carcinoma | 0.208 | 7.44E-08 | *TGFB2, TGFB3, PHF10, PLCG1, AKT3, SMARCE1, FZD1, FZD4, AXIN2, FZD7, GADD45G, DVL3, PIK3R2, PIK3R1, TERT, IGF2, TGFBR2, TGFBR1, SMARCB1, SHC2, PRKCB, SMARCD2, HGF, LEF1, ACTB, KRAS, LRP6, LRP5, WNT2, TCF7L1, TCF7L2, MET, MGST1, WNT5B, WNT5A* |
| Colorectal cancer | 0.279 | 9.19E-08 | *TGFB2, TGFB3, PMAIP1, AKT3, LEF1, AXIN2, JUN, MLH1, GADD45G, PIK3R2, PIK3R1, MAPK8, TGFBR2, TGFBR1, RAC2, RALGDS, APPL1, HGF, RHOA, KRAS, CASP3, TCF7L1, TCF7L2, BCL2* |
| Wnt signaling pathway | 0.206 | 2.11E-07 | *RYK, CTNND2, CER1, VANGL1, LEF1, FZD1, PRICKLE2, FZD4, AXIN2, FZD7, WISP1, JUN, CBY1, DVL3, MAPK8, RAC2, CAMK2D, DAAM1, PRKCB, BAMBI, SERPINF1, RHOA, CSNK2A2, RSPO3, LRP6, ZNRF3, LRP5, WNT2, CCND2, TCF7L1, TCF7L2, WNT5B, WNT5A* |
| Apoptosis | 0.221 | 2.15E-07 | *NGF, TNFSF10, PMAIP1, AKT3, LAMB2, BID, JUN, GADD45G, PIK3R2, PIK3R1, MAPK8, ERN1, CAPN2, TRAF1, CTSK, DDIT3, DFFA, PARP3, CTSZ, CTSS, KRAS, LMNB2, LMNB1, CASP2, CASP3, TNFRSF1A, PTPN13, CASP6, ACTB, BCL2* |
| Cell cycle | 0.226 | 3.59E-07 | *HDAC1, TGFB2, HDAC2, BUB1B, PLK1, MAD2L1, ABL1, CCNA2, SMC3, YWHAQ, GADD45G, TTK, PTTG1, TGFB3, BUB1, BUB3, ORC3, ORC1, MCM5, MCM4, MCM3, ANAPC4, CCNB3, CCND2, CDC20, CDK1, DBF4, CCNH* |
| Breast cancer | 0.204 | 8.94E-07 | *HGF, AKT3, LEF1, FZD1, FZD4, AXIN2, FZD7, JUN, GADD45G, DVL3, FGF18, PGR, PIK3R2, PIK3R1, JAG2, FGF10, IGF1, SHC2, FGF7, KRAS, FGF2, FGF1, FGFR1, LRP6, LRP5, WNT2, TCF7L1, TCF7L2, WNT5B, WNT5A* |
| mTOR signaling pathway | 0.190 | 4.81E-06 | *SLC7A5, PRKAA1, AKT3, RICTOR, TSC2, NPRL3, FZD1, FZD4, FZD7, PRR5, DVL3, PIK3R2, PIK3R1, RNF152, IGF1, SEC13, PRKCB, WNT5B, HGF, RHOA, KRAS, CAB39L, LRP6, RPS6KA6, TNFRSF1A, WNT2, LRP5, ATP6V1C2, WNT5A* |
| Basal cell carcinoma | 0.270 | 9.68E-06 | *FZD1, FZD4, BMP2, FZD7, WNT2, TCF7L1, TCF7L2, GADD45G, GLI2, DVL3, WNT5B, AXIN2, WNT5A, PTCH1, LEF1, SMO, HHIP* |
| p53 signaling pathway | 0.250 | 1.30E-05 | *CD82, PMAIP1, CASP3, SESN3, MDM4, BID, CCND2, TP53I3, THBS1, CDK1, CCNG2, IGF1, RRM2, STEAP3, GADD45G, TSC2, IGFBP3, BCL2* |
| Amino sugar and nucleotide sugar metabolism | 0.292 | 2.82E-05 | *GALT, UAP1, GALK1, HEXA, GALE, UGDH, HEXB, GMPPB, TSTA3, GMDS, UAP1L1, GNE, GFPT2, FUK* |
| DNA replication | 0.333 | 3.55E-05 | *POLA2, RFC5, POLE, RFC3, RFC2, MCM5, MCM4, MCM3, POLE2, PRIM2, POLE3, RPA2* |
| EGFR tyrosine kinase inhibitor resistance | 0.228 | 3.77E-05 | *SRC, PDGFRA, PDGFC, NF1, SHC2, PDGFA, PRKCB, NRG1, MET, PLCG1, AKT3, PIK3R2, PIK3R1, HGF, IGF1, KRAS, FGF2, BCL2* |
| Oocyte meiosis | 0.172 | 2.21E-04 | *MAD2L1, PLK1, CDC20, ADCY2, SMC3, YWHAQ, PGR, AURKA, PTTG1, IGF1, BUB1, CAMK2D, SGOL1, ANAPC4, PPP1CC, RPS6KA6, MOS, PPP2CB, CALML4, CDK1, PPP2R5D, PPP2R5C* |
| Relaxin signaling pathway | 0.169 | 2.68E-04 | *TGFBR2, AKT3, COL3A1, RXFP3, JUN, GNG12, PIK3R2, PIK3R1, MAPK8, SRC, TGFBR1, SHC2, CREB5, COL4A6, COL4A5, HGF, KRAS, ADCY2, RLF, CREB3L1, COL1A2, MMP2* |
| Purine metabolism | 0.169 | 2.68E-04 | *CECR1, PDE9A, RRM2, PAPSS2, DCK, PDE1A, NT5C3B, PDE7A, PDE10A, NPR1, NPR2, ENTPD2, ENTPD1, NUDT5, APRT, ADK, GMPS, ATIC, ADCY2, PDE6D, DGUOK, PDE8A* |
| Cellular senescence | 0.156 | 3.16E-04 | *TGFB2, TGFB3, RASSF5, AKT3, FOXM1, VDAC3, TSC2, CCNA2, GADD45G, PIK3R2, PIK3R1, CAPN2, TGFBR2, TGFBR1, ZFP36L1, SLC25A6, IGFBP3, KRAS, CCNB3, MRAS, PPP1CC, CCND2, CALML4, CDK1, LIN52* |
| ErbB signaling pathway | 0.188 | 6.48E-04 | *SRC, NRG1, SHC2, CAMK2D, PRKCB, JUN, ABL1, PLCG1, AKT3, PIK3R2, PIK3R1, HGF, MAPK8, ABL2, NRG3, KRAS* |
| FoxO signaling pathway | 0.159 | 7.47E-04 | *CCNB3, TGFBR2, TGFBR1, TNFSF10, KRAS, PLK1, PLK3, CCND2, TGFB2, PRKAA1, PLK4, MST1, CCNG2, AKT3, PIK3R2, PIK3R1, HGF, MAPK8, GADD45G, TGFB3, IGF1* |
| Prostate cancer | 0.175 | 9.05E-04 | *PDGFA, PDGFRA, PDGFC, CREB5, IGF1, TCF7L1, TCF7L2, AKT3, CREB3L1, PIK3R2, PIK3R1, HGF, KRAS, FGFR1, ZEB1, LEF1, BCL2* |
| Endometrial cancer | 0.207 | 1.48E-03 | *AXIN2, MLH1, TCF7L1, TCF7L2, ILK, AKT3, PIK3R2, PIK3R1, HGF, LEF1, GADD45G, KRAS* |
| Small cell lung cancer | 0.172 | 1.50E-03 | *TRAF1, LAMA2, TRAF3, TRAF4, CASP3, FN1, ITGAV, GADD45G, COL4A6, COL4A5, AKT3, PIK3R2, PIK3R1, LAMB1, LAMC1, BCL2* |
| Hippo signaling pathway-multiple species | 0.276 | 1.97E-03 | *WWTR1, RASSF2, RASSF6, LATS2, FAT4, FRMD6, FRMD1, YAP1* |
| Mismatch repair | 0.304 | 2.36E-03 | *RFC5, EXO1, MLH1, RFC2, RFC3, PMS2, RPA2* |
| Viral carcinogenesis | 0.129 | 2.84E-03 | *HDAC1, HDAC2, PMAIP1, TRAF1, VDAC3, GSN, CCNA2, CHD4, RBPJ, YWHAQ, JUN, PIK3R2, PIK3R1, CREB5, SRC, TRAF3, GTF2H1, RHOA, RASA2, KRAS, CASP3, CCND2, CDC20, CDK1, CREB3L1, HDAC11* |
| Nucleotide excision repair | 0.213 | 2.99E-03 | *RFC5, GTF2H1, RFC3, RFC2, POLE, POLE3, POLE2, RPA2, CCNH, XPA* |
| Pyrimidine metabolism | 0.193 | 3.66E-03 | *DCK, ENTPD1, DPYD, CMPK1, DCTD, NT5C3B, TYMS, RRM2, CAD, DUT, CTPS2* |
| Pancreatic cancer | 0.173 | 3.77E-03 | *TGFBR2, TGFBR1, TGFB3, RAC2, PLD1, TGFB2, GADD45G, AKT3, PIK3R2, PIK3R1, MAPK8, KRAS, RALGDS* |
| Homologous recombination | 0.220 | 3.98E-03 | *ECD, RAD54B, BARD1, RAD54L, FAM175A, RPA2, RAD52, UIMC1, TOP3B* |
| Jak-STAT signaling pathway | 0.130 | 6.66E-03 | *SOCS5, PDGFA, PDGFRA, THPO, AKT3, SOCS2, STAM2, CCND2, SOCS3, LEPR, PIAS2, CNTFR, PIAS1, CISH, PIK3R1, IL17D, HGF, BCL2, STAM, PTPN6, PIK3R2* |
| Notch signaling pathway | 0.188 | 9.60E-03 | *HDAC1, APH1A, HDAC2, RBPJ, NCSTN, DVL3, NUMB, JAG2, NCOR2* |
| VEGF signaling pathway | 0.169 | 1.18E-02 | *SRC, RAC2, SHC2, PRKCB, PLCG1, AKT3, PIK3R2, PIK3R1, PLA2G4A, KRAS* |
| Autophagy-animal | 0.125 | 2.16E-02 | *KRAS, PIK3R1, TSC2, AMBRA1, PPP2CB, EIF2AK4, PRKAA1, MRAS, AKT3, LAMP2, LAMP1, MAPK8, BCL2, DAPK3, ERN1, PIK3R2* |
| Non-small cell lung cancer | 0.152 | 2.23E-02 | *PRKCB, RASSF5, MST1, GADD45G, PLCG1, AKT3, PIK3R2, PIK3R1, HGF, KRAS* |
| Renal cell carcinoma | 0.145 | 2.83E-02 | *TGFB2, TGFB3, JUN, MET, AKT3, PIK3R2, PIK3R1, HGF, HLF, KRAS* |
| Ferroptosis | 0.175 | 2.84E-02 | *SAT1, GSS, ACSL3, VDAC3, MAP1LC3C, FTH1, STEAP3* |
| Apoptosis-multiple species | 0.182 | 3.57E-02 | *PMAIP1, CASP3, TNFRSF1A, BID, MAPK8, BCL2* |
| Base excision repair | 0.182 | 3.57E-02 | *MPG, TDG, PARP3, POLE, POLE3, POLE2* |
| Mitophagy-animal | 0.138 | 4.53E-02 | *SRC, TBK1, AMBRA1, JUN, MRAS, CITED2, MAPK8, CSNK2A2, KRAS* |
| **Immune response** | | | |
| Focal adhesion | 0.261 | 3.69E-14 | *ITGA9, PDGFRA, CCND2, CHAD, PDGFA, TNXB, ILK, ITGB3, ITGA11, BCL2, LAMB1, THBS1, COL2A1, CAV3, ZYX, THBS2, FYN, THBS3, TLN1, RELN, PIK3R1, MAPK8, PIK3R2, SRC, LAMA2, PDGFC, RAC2, SHC2, PRKCB, MYL10, COL4A6, COL4A5, JUN, TNC, HGF, RHOA, LAMC1, ACTB, PPP1CC, FN1, VAV2, CAPN2, ITGAV, MET, AKT3, COL1A2, IGF1, COL6A1, TLN2, COL6A3, COL6A2, COL6A6* |
| Human papillomavirus infection | 0.191 | 1.49E-11 | *HDAC1, ITGA9, HDAC2, VMA21, CHAD, THBS2, COL4A6, ITGB3, ITGA11, ITGAV, IKBKE, LAMB1, TSC2, FZD1, DLG3, FZD4, AXIN2, TBK1, FZD7, PTGER4, COL2A1, RBPJ, TNXB, THBS3, THBS1, ATP6V0E1, DVL3, RELN, PIK3R1, TERT, PIK3R2, PPP2CB, LAMA2, TRAF3, TCIRG1, NFX1, CREB5, COL6A1, COL4A5, TNC, ATP6V1C2, HGF, CCNA2, KRAS, LAMC1, COL6A3, CHD4, COL6A2, CASP3, FN1, TNFRSF1A, WNT2, CCND2, TCF7L1, TCF7L2, AKT3, CREB3L1, COL1A2, PPP2R5D, WNT5B, WNT5A, PPP2R5C, COL6A6* |
| Human T-cell leukemia virus 1 infection | 0.164 | 6.64E-06 | *TGFB2, MAD2L1, BUB1B, TLN2, NFYB, AKT3, VDAC3, CANX, SPI1, CCNA2, MAP3K3, EGR1, JUN, TLN1, ETS2, PIK3R2, PIK3R1, PTTG1, MAPK8, TERT, TGFB3, TGFBR2, TGFBR1, ITGB2, BUB3, CREB5, SLC25A6, TRRAP, ANAPC4, KRAS, ADCY2, TNFRSF1A, CCND2, CDC20, CREB3L1, CD3D* |
| Fc gamma R-mediated phagocytosis | 0.223 | 1.14E-05 | *MARCKSL1, NCF1, LIMK1, RAC2, PLD1, INPPL1, ARPC3, ARPC1B, VAV2, ARPC5, DOCK2, PRKCB, PLCG1, AKT3, PIK3R2, PIK3R1, INPP5D, PLA2G4A, MYO10, PPAP2B, GSN* |
| Adherens junction | 0.250 | 1.30E-05 | *SRC, TGFBR1, RAC2, ACP1, ACTB, MET, RHOA, FYN, TCF7L1, TCF7L2, PTPN6, PTPRF, TGFBR2, PTPN1, LEF1, CSNK2A2, YES1, FGFR1* |
| Phagosome | 0.178 | 2.77E-05 | *SFTPD, NCF1, LAMP1, NCF4, ITGB2, CANX, MRC2, THBS2, THBS3, THBS1, TUBB6, ITGB3, ATP6V0E1, PLA2R1, LAMP2, VMA21, C1R, CTSS, DYNC2H1, SEC22B, CLP1, ITGAV, TCIRG1, COLEC12, ATP6V1C2, ACTB, SEC61A1* |
| Hepatitis B | 0.172 | 3.37E-05 | *TGFB2, TGFB3, TGFBR2, PTK2B, AKT3, IKBKE, VDAC3, CCNA2, TBK1, BID, YWHAQ, JUN, PIK3R2, PIK3R1, MAPK8, MYD88, IRAK4, SRC, TGFBR1, TRAF3, CREB5, PRKCB, HGF, KRAS, CASP3, CREB3L1, MAVS, BCL2* |
| Cell adhesion molecules (CAMs) | 0.178 | 3.72E-05 | *ITGA9, CLDN11, VCAN, GLG1, CNTN1, ITGAV, CD2, ICOSLG, NCAM1, NTNG1, CD80, NEO1, CLDN1, CDH2, CADM1, ITGB2, JAM3, NEGR1, F3, LRRC4B, CD99, CD276, NRXN1, PTPRF, ALCAM, SIGLEC1* |
| Bacterial invasion of epithelial cells | 0.230 | 5.62E-05 | *SRC, RHOA, PIK3R1, FN1, ARPC3, ARPC1B, DNM3, ILK, ARPC5, CAV3, MET, DNM1, PIK3R2, ARHGAP10, ACTB, SHC2, ELMO1* |
| Leukocyte transendothelial migration | 0.188 | 1.06E-04 | *NCF1, CLDN11, RAC2, CD99, RHOA, MYL10, VAV2, RASSF5, PLCG1, JAM3, NCF4, PRKCB, PTK2B, ITGB2, PIK3R2, PIK3R1, CLDN1, THY1, CXCL12, MMP2, ACTB* |
| Yersinia infection | 0.182 | 1.09E-04 | *SKAP2, LIMK1, PLCG1, AKT3, FYB, TBK1, ARHGEF7, JUN, PIK3R2, PIK3R1, MAPK8, MYD88, IRAK4, SRC, RAC2, RHOA, RPS6KA6, FN1, VAV2, PTK2B, GIT2, ACTB* |
| Melanoma | 0.222 | 1.28E-04 | *PDGFA, PDGFRA, PDGFC, IGF1, GADD45G, MET, AKT3, PIK3R2, PIK3R1, FGF7, FGF18, KRAS, FGF10, FGF2, FGF1, FGFR1* |
| Tuberculosis | 0.151 | 3.08E-04 | *CD74, TGFB2, TGFB3, PLK3, NFYB, NFYA, AKT3, MRC2, BID, CAMK2D, MAPK8, MYD88, IRAK4, SRC, RFXANK, ITGB2, PLA2R1, LAMP2, LAMP1, RHOA, CTSS, CASP3, TNFRSF1A, LSP1, CALML4, TCIRG1, BCL2* |
| Hepatitis C | 0.155 | 4.63E-04 | *CLDN11, AKT3, IKBKE, TBK1, CD81, BID, YWHAQ, EIF3E, PIK3R2, PIK3R1, CLDN1, RNASEL, TRAF3, PPARA, HGF, KRAS, CASP3, SOCS3, TNFRSF1A, EIF2AK1, PPP2CB, EIF2AK4, PIAS1, MAVS* |
| Fc epsilon RI signaling pathway | 0.206 | 6.44E-04 | *RAC2, ALOX5, VAV2, FYN, PLCG1, AKT3, PIK3R2, PIK3R1, HGF, INPP5D, PLA2G4A, ALOX5AP, MAPK8, KRAS* |
| Human cytomegalovirus infection | 0.133 | 9.29E-04 | *PDGFRA, PTK2B, AKT3, GNG12, TSC2, TMEM173, TBK1, PTGER4, BID, PTGER2, PIK3R2, PIK3R1, SRC, ITGB3, RAC2, CREB5, PRKCB, PDIA3, HGF, RHOA, CXCL12, KRAS, CASP3, ADCY2, TNFRSF1A, ITGAV, CALML4, CREB3L1, GNA11, AKAP13* |
| Pathogenic Escherichia coli infection | 0.218 | 9.92E-04 | *TUBB6, ARPC1B, ARPC3, YWHAQ, FYN, ARPC5, LY96, NCL, ABL1, CLDN1, ACTB, RHOA* |
| Chemokine signaling pathway | 0.137 | 1.41E-03 | *NCF1, DOCK2, PTK2B, AKT3, ADRBK2, PREX1, GRK4, GRK5, XCR1, GNG12, TIAM1, PIK3R2, PIK3R1, SRC, RAC2, SHC2, PRKCB, HGF, RHOA, CXCL12, CXCL14, KRAS, ADCY2, CX3CR1, VAV2, ELMO1* |
| Epstein-Barr virus infection | 0.134 | 1.47E-03 | *HDAC1, RPN2, HDAC2, AKT3, IKBKE, NCOR2, ECD, CCNA2, TBK1, RBPJ, JUN, GADD45G, PIK3R2, PIK3R1, MAPK8, MYD88, IRAK4, TRAF3, ENTPD1, PDIA3, CASP3, CCND2, VIM, MAVS, CD3D, BID, BCL2* |
| Human immunodeficiency virus 1 infection | 0.132 | 1.53E-03 | *LIMK1, PLCG1, AKT3, TMEM173, TBK1, BID, JUN, GNG12, PIK3R2, PIK3R1, MAPK8, MYD88, IRAK4, RAC2, PRKCB, PDIA3, KRAS, CCNB3, SAMHD1, CASP3, TNFRSF1A, AP1G1, PTK2B, CALML4, CDK1, GNA11, CD3D, BCL2* |
| Amoebiasis | 0.168 | 1.82E-03 | *LAMA2, TGFB3, CASP3, FN1, PRKCB, TGFB2, COL4A6, COL4A5, ITGB2, COL1A2, PIK3R2, PIK3R1, GNA11, LAMB1, COL3A1, LAMC1* |
| Platelet activation | 0.153 | 1.91E-03 | *SRC, ITGB3, APBB1IP, PPP1CC, ADCY2, TBXA2R, RHOA, TLN2, SNAP23, FYN, P2RY12, TLN1, AKT3, COL1A2, PIK3R2, PIK3R1, COL3A1, PLA2G4A, ACTB* |
| Complement and coagulation cascades | 0.177 | 2.26E-03 | *TFPI, ITGB2, F5, CFH, VSIG4, SERPINF2, PROS1, MASP2, SERPING1, C1R, C1S, F13A1, C7, SERPIND1* |
| Measles | 0.145 | 2.64E-03 | *EIF3H, TRAF3, MYD88, CASP3, TBK1, EIF2AK1, BID, CCND2, JUN, EIF2AK4, AKT3, PIK3R2, PIK3R1, IKBKE, MAPK8, MAVS, CD3D, CSNK2A2, IRAK4, BCL2* |
| B cell receptor signaling pathway | 0.171 | 3.05E-03 | *PRKCB, RAC2, INPPL1, CD81, CARD11, VAV2, JUN, AKT3, PIK3R2, PIK3R1, HGF, INPP5D, PTPN6, KRAS* |
| Melanogenesis | 0.158 | 3.13E-03 | *FZD1, FZD4, ADCY2, FZD7, CAMK2D, WNT2, PRKCB, TCF7L1, TCF7L2, CALML4, DVL3, CREB3L1, WNT5B, WNT5A, LEF1, KRAS* |
| Natural killer cell mediated cytotoxicity | 0.145 | 3.30E-03 | *TNFSF10, RAC2, CASP3, SHC2, SH2D1B, BID, VAV2, FYN, PLCG1, MTCP1, SH3BP2, PTK2B, ITGB2, PIK3R2, PIK3R1, HGF, PRKCB, PTPN6, KRAS* |
| Chagas disease (American trypanosomiasis) | 0.155 | 3.71E-03 | *TGFBR2, TGFBR1, TGFB3, ACE, TNFRSF1A, PPP2CB, JUN, TGFB2, AKT3, PIK3R2, PIK3R1, GNA11, MAPK8, MYD88, CD3D, IRAK4* |
| Malaria | 0.204 | 3.88E-03 | *TGFB2, LRP1, CD81, THBS2, THBS3, ITGB2, THBS1, MET, TGFB3, MYD88* |
| Pertussis | 0.171 | 4.16E-03 | *ITGB2, CASP3, JUN, CALML4, C1S, SERPING1, NOD1, LY96, C1R, MAPK8, MYD88, RHOA, IRAK4* |
| Vibrio cholerae infection | 0.200 | 4.40E-03 | *ATP6V0E1, PDIA4, TCIRG1, PLCG1, VMA21, ATP6V1C2, KDELR2, KDELR3, ACTB, SEC61A1* |
| Viral myocarditis | 0.183 | 5.14E-03 | *LAMA2, ITGB2, RAC2, CASP3, CD80, BID, FYN, ABL1, DMD, ACTB, ABL2* |
| Epithelial cell signaling in Helicobacter pylori infection | 0.171 | 5.69E-03 | *SRC, CASP3, JAM3, JUN, TCIRG1, MET, PLCG1, NOD1, VMA21, ATP6V0E1, ATP6V1C2, MAPK8* |
| Inflammatory mediator regulation of TRP channels | 0.150 | 6.45E-03 | *SRC, NGF, IGF1, PPP1CC, ADCY2, CAMK2D, PRKCB, PTGER2, CALML4, PTGER4, PLCG1, PIK3R2, PIK3R1, PLA2G4A, MAPK8* |
| C-type lectin receptor signaling pathway | 0.144 | 8.80E-03 | *IL17D, SRC, PLK3, LSP1, JUN, MRAS, CALML4, AKT3, CYLD, PIK3R2, PIK3R1, IKBKE, MAPK8, RHOA, KRAS* |
| Influenza A | 0.126 | 8.97E-03 | *TNFSF10, TRAF3, MYD88, CASP3, TBK1, TNFRSF1A, BID, PRKCB, SLC25A6, SOCS3, AKT3, KPNA2, PIK3R1, IKBKE, PML, ACTB, MAVS, NLRX1, RNASEL, IRAK4, PIK3R2* |
| Cytokine-cytokine receptor interaction | 0.109 | 1.05E-02 | *NGF, TNFSF10, ACVR1, ACVR2A, CSF1R, ACVR2B, BMPR1A, BMPR1B, TGFB3, XCR1, THPO, INHBA, GDF10, IL17D, TGFBR2, TGFBR1, IL17A, IL18R1, LEPR, CNTFR, CXCL12, CXCL14, TGFB2, BMP7, BMP6, BMP2, CX3CR1, TNFRSF1A, CSF1, ACKR3, BMPR2, IL17RA* |
| Gap junction | 0.148 | 1.20E-02 | *SRC, PDGFRA, PDGFC, ADCY2, PDGFA, TUBB6, PRKCB, CDK1, MAP2K5, GNA11, HGF, LPAR1, KRAS* |
| Rheumatoid arthritis | 0.143 | 1.50E-02 | *TGFB2, TGFB3, IL17A, CD80, CTSK, JUN, CSF1, TCIRG1, ITGB2, VMA21, ATP6V0E1, ATP6V1C2, CXCL12* |
| TNF signaling pathway | 0.134 | 1.55E-02 | *TRAF1, TRAF3, CASP3, SOCS3, TNFRSF1A, CREB5, JUN, CSF1, IL18R1, AKT3, CREB3L1, PIK3R2, PIK3R1, MAPK8, RPS6KA5* |
| NOD-like receptor signaling pathway | 0.118 | 1.63E-02 | *PAN3, NEK7, TRAF3, TANK, MYD88, TBK1, ANTXR2, RHOA, JUN, RNASEL, NOD1, IRAK4, IKBKE, VDAC3, PANX1, MAPK8, MAVS, NLRX1, TMEM173, PSTPIP1, BCL2* |
| T cell receptor signaling pathway | 0.136 | 1.70E-02 | *RHOA, JUN, CARD11, VAV2, FYN, PLCG1, AKT3, PIK3R2, PIK3R1, HGF, MAPK8, CD3D, PTPN6, KRAS* |
| Leishmaniasis | 0.149 | 1.91E-02 | *MARCKSL1, NCF1, TGFB3, NCF4, PRKCB, JUN, TGFB2, ITGB2, MYD88, PTPN6, IRAK4* |
| Shigellosis | 0.147 | 2.62E-02 | *SRC, ACTB, ARPC3, ARPC1B, ARPC5, UBE2D1, NOD1, ABL1, MAPK8, ELMO1* |
| NF-kappa B signaling pathway | 0.130 | 2.80E-02 | *TRAF1, TRAF3, TNFRSF1A, CARD11, PRKCB, PLCG1, CXCL12, CYLD, LY96, MYD88, CSNK2A2, IRAK4, BCL2* |
| Toxoplasmosis | 0.124 | 3.20E-02 | *LAMA2, LAMC1, CASP3, TNFRSF1A, ALOX5, TGFB2, LAMB1, AKT3, LY96, MAPK8, MYD88, TGFB3, IRAK4, BCL2* |
| Toll-like receptor signaling pathway | 0.125 | 3.58E-02 | *CTSK, TRAF3, TBK1, CD80, JUN, AKT3, PIK3R2, PIK3R1, IKBKE, LY96, MAPK8, MYD88, IRAK4* |
| **Others** | | | |
| Metabolic pathways | 0.152 | 5.86E-25 | *DOLK, PPAP2B, TGDS, GALC, MAT1A, CHSY1, MAN1B1, GAMT, DMGDH, GCAT, PAH, PYCR1, HPSE2, CERS2, SGPL1, CERS5, AHCYL1, DCK, B3GNT2, DHRS9, PPT1, DNMT1, PDE10A, PDE7A, ENTPD1, CAD, SPTLC1, ASRGL1, PAFAH1B2, PYGL, NPR1, NPR2, ENTPD2, EXT1, HOGA1, ALOX5, XYLT2, XYLT1, APRT, PDE8A, CNDP2, GMPS, PLD4, P4HA2, ATIC, INPP5D, PLD1, ADCY2, ACSL3, EPT1, ETNPPL, OAT, BST1, MMAB, DBT, HSD17B12, ALG8, PDXK, AHCY, TRAK2, ALG1, ALG6, H6PD, ALG5, LARGE, MGST1, TPK1, CDO1, CHKA, PANK3, GALNT5, DCTD, GATM, TKT, GMDS, B4GALT1, B4GALT3, B4GALT2, PTS, B4GALT7, UAP1, MTAP, MTHFD2L, NUDT5, CHST9, RIMKLB, INPPL1, CHDH, STT3A, DSE, CSGALNACT2, MCCC2, MCCC1, CSGALNACT1, CHAC1, HACD4, GAB1, PIGS, TCIRG1, GPX3, SARDH, ELOVL2, PDE6D, POMT1, GPX8, GFPT2, B4GALNT4, AGPS, ACP1, PI4KA, GNS, UGT8, LCLAT1, PIGW, IDS, MBOAT2, MBOAT1, HGSNAT, ME3, STT3B, PTGES, COASY, CTPS2, IPPK, PYGB, NT5C3B, AMDHD1, HEXDC, ALDH18A1, DPM2, ASL, GALT, CECR1, CA5A, ATP6V0E1, ACACA, WBSCR17, UGDH, NAGLU, GMPPB, GALE, UAP1L1, PLCD3, ALDH7A1, ALG11, MVK, DUT, ADK, GALK1, GALNT16, TRIT1, ADH5, TSTA3, GPT2, PDE9A, OXCT1, DGUOK, OCRL, SAT1, CBLB, PDXP, PLCG1, ATP6V1C2, RRM2, ABAT, GNE, ALDH1A2, GYG2, GCNT4, PAPSS2, SHPK, RDH8, DPYD, AASS, CMPK1, PDE1A, NAPRT, FUK, ACSS3, C1GALT1, ELOVL5, ETNK2, SYNJ2, ALDH1A3, SEPHS1, HSD17B7, ST3GAL1, AADAT, GPX7, ST3GAL4, PIGH, GALNS, PIGM, PIGP, MGAT1, MAN1C1, VMA21, NAGA, ST6GALNAC6, PLA2G4A, BDH2, CHST10, CA8, ASAH2, ARSB, ENO1, HEXA, HEXB, AKR1D1, EXT2, PSAT1, TYMS, RDH10, A4GALT, CHPF, FAP, ESD, ALDH9A1* |
| Axon guidance | 0.287 | 1.49E-15 | *RYK, BMP7, EPHB3, EPHA6, LIMK1, EPHA3, ILK, SRGAP3, SRGAP2, SMO, BMPR1B, TRPC1, TRPC6, ABL1, SEMA4A, SEMA6D, BOC, SEMA4B, SEMA6A, PLXNB2, NTNG1, FYN, CAMK2D, NEO1, SLIT3, PIK3R2, PIK3R1, PLXNC1, KRAS, EPHB2, SRC, EPHB1, RAC2, UNC5B, EFNA5, ABLIM3, RHOA, CXCL12, RASA1, SEMA3D, SEMA3A, SEMA3C, SEMA5B, SEMA5A, SEMA3F, ROBO1, PLCG1, MET, BMPR2, WNT5B, WNT5A, PTCH1* |
| Ribosome | 0.275 | 2.65E-12 | *RPS13, RPS12, RPS11, RPS17, RPS16, RPL8, RPL9, RPL27A, RPS27A, RPL18A, RPL35A, RPS15A, RPS7, RPS25, RPS8, RPL22L1, RPL27, RPL21, RPL23, RPL10A, RPL4, RPS24, MRPL35, MRPL32, RPS23, RPS21, RPL14, RPL15, RPL17, RPL12, RPL13, RPS4X, RPL19, RPSA, RPL39, RPL34, RPL35, RPL32, RPL30, RPL31, RPS3A, NOV* |
| Proteoglycans in cancer | 0.232 | 2.26E-11 | *TGFB2, LUM, PPP1CC, SMO, PTPN6, AKT3, HPSE2, CAV3, DCN, FZD1, DROSHA, FZD4, ACTB, FZD7, TWIST1, TWIST2, THBS1, TIAM1, PIK3R2, PIK3R1, PDCD4, KRAS, IGF2, SRC, ITGB3, IGF1, CAMK2D, PRKCB, TFAP4, MRAS, GPC1, HGF, RHOA, FGF2, FGFR1, CASP3, FN1, WNT2, VAV2, PLCG1, ITGAV, MET, COL21A1, WNT5B, WNT5A, PTCH1, MMP2* |
| Lysosome | 0.276 | 2.63E-10 | *GNS, NAGPA, GM2A, GNPTAB, NAGA, IDS, LAPTM5, AP3S1, HGSNAT, DNASE2B, PPT1, NPC2, CTSA, AP3B1, CTSK, TPP1, NAGLU, GALNS, CTSZ, LAMP2, LAMP1, GALC, CTSS, CTNS, ARSG, ARSB, HEXA, SCARB2, HEXB, CD164, TCIRG1, ABCA2, AP1G1, FUCA1* |
| Protein processing in endoplasmic reticulum | 0.224 | 6.05E-09 | *UBE2J1, SEC63, MAN1B1, MBTPS2, CANX, DNAJB11, DERL3, CAPN2, DNAJC10, RRBP1, UGGT2, ERLEC1, ERN1, UGGT1, DDIT3, PDIA6, SEC13, PDIA4, PDIA3, SEC24D, MAN1C1, LMAN1, EDEM3, SEC24B, STT3B, STT3A, UBE2D1, MAPK8, P4HB, MBTPS1, EIF2AK1, EIF2AK4, TRAM1, SSR1, SSR3, SEC61A1, BCL2* |
| Regulation of actin cytoskeleton | 0.201 | 6.51E-09 | *MYH10, ITGA9, PDGFRA, PDGFA, LIMK1, HGF, ITGA11, FGFR4, IQGAP2, GSN, ARHGEF7, CYFIP2, GNG12, TIAM1, FGF18, PIK3R2, PIK3R1, FGF10, SRC, ITGB3, PDGFC, RAC2, ARPC3, MYL10, MRAS, ARPC5, FGF7, RHOA, CXCL12, KRAS, FGF2, FGF1, FGFR1, PPP1CC, FN1, MOS, ARPC1B, VAV2, ITGAV, ITGB2, LPAR2, ACTB, LPAR1* |
| Endocytosis | 0.180 | 6.99E-08 | *SNX2, SNX1, SNX6, TGFBR2, PDGFRA, DNM1, ARFGAP3, DNM3, KIAA1033, DAB2, STAM, CAV3, SH3GL3, SMURF1, SMAP1, RAB8A, RAB31, STAM2, GRK5, ZFYVE16, PSD3, ARPC3, RAB22A, ADRBK2, PML, SRC, TGFBR1, CCDC53, KIAA0196, RUFY1, ARPC5, AGAP1, SMURF2, ACAP2, EPS15L1, ARAP1, RHOA, CYTH4, RAB11FIP2, PLD1, ARPC1B, FGFR4, GIT2, GRK4* |
| Signaling pathways regulating pluripotency of stem cells | 0.214 | 3.68E-07 | *ACVR1, MYF5, BMPR1A, BMPR1B, AKT3, INHBA, FZD1, FZD4, AXIN2, FZD7, DVL3, KLF4, PIK3R2, PIK3R1, KRAS, ACVR2A, ACVR2B, IGF1, REST, SKIL, FGFR4, FGF2, FGFR1, PCGF3, PCGF6, WNT2, ID2, BMPR2, WNT5B, WNT5A* |
| Fluid shear stress and atherosclerosis | 0.209 | 9.16E-07 | *NCF1, ACVR1, PDGFA, ITGB3, PRKAA1, BMPR1A, BMPR1B, AKT3, MAP2K5, CAV3, JUN, PIK3R2, PIK3R1, MAPK8, SRC, ACVR2B, RAC2, CNP, GPC1, RHOA, TNFRSF1A, ITGAV, CALML4, MGST1, BMPR2, ACTB, MMP2, ACVR2A, BCL2* |
| Neurotrophin signaling pathway | 0.210 | 4.52E-06 | *NGF, PLCG1, AKT3, MAP2K5, ABL1, BDNF, MAP3K3, JUN, CAMK4, NTRK2, NTRK3, ARHGDIB, PIK3R2, PIK3R1, MAPK8, IRAK4, SHC2, CAMK2D, HGF, RHOA, KRAS, RPS6KA5, RPS6KA6, CALML4, BCL2* |
| Protein digestion and absorption | 0.233 | 6.45E-06 | *COL15A1, COL11A1, COL4A6, COL18A1, KCNK5, COL22A1, COL14A1, KCNE3, COL5A2, COL6A1, COL4A5, COL13A1, COL1A2, COL2A1, COL21A1, COL3A1, COL6A3, COL6A2, DPP4, COL12A1, COL6A6* |
| cAMP signaling pathway | 0.164 | 9.75E-06 | *AKT3, BDNF, SOX9, JUN, CAMK4, PTGER2, GRIN3A, TIAM1, VIPR2, PIK3R2, PIK3R1, GLP1R, EDN3, EDN2, NPR1, RAC2, CAMK2D, CREB5, MAPK8, HTR6, HTR4, PPARA, RHOA, PDE10A, PPP1CC, PLD1, ADCY2, VAV2, CALML4, CREB3L1, OXTR, PLN, PTCH1, HTR1A, HHIP* |
| Hedgehog signaling pathway | 0.298 | 2.32E-05 | *SMURF1, SMO, SMURF2, BOC, EVC, EVC2, GLI2, CDON, CCND2, ADRBK2, BCL2, PTCH1, KIF3A, HHIP* |
| Transcriptional misregulation in cancer | 0.161 | 5.09E-05 | *HDAC1, HDAC2, PDGFA, SCG2, IGF1, CCNT1, CCNT2, HPGD, MYCN, SPI1, CSF1R, CDK14, SUPT3H, KDM6A, H3F3B, PML, KLF3, TGFBR2, TRAF1, DDIT3, IGFBP3, PAX7, ZEB1, ETV1, GADD45G, CCND2, ID2, MET, RUNX1, MAF* |
| Arginine and proline metabolism | 0.240 | 4.80E-04 | *SAT1, GAMT, L3HYPDH, P4HA2, GATM, OAT, ALDH9A1, ALDH7A1, HOGA1, PYCR1, ALDH18A1, CNDP2* |
| Osteoclast differentiation | 0.164 | 5.25E-04 | *TGFBR2, NCF1, SPI1, SOCS3, TNFRSF1A, FYN, CALCR, NCF4, JUN, TGFBR1, CAMK4, ITGB3, AKT3, CYLD, PIK3R2, PIK3R1, CTSK, CSF1R, MAPK8, TGFB2, CSF1* |
| Glioma | 0.200 | 5.47E-04 | *PDGFA, PDGFRA, IGF1, SHC2, CAMK2D, PRKCB, CAMK4, GADD45G, PLCG1, AKT3, PIK3R2, PIK3R1, HGF, CALML4, KRAS* |
| Chronic myeloid leukemia | 0.197 | 6.17E-04 | *HDAC1, TGFBR2, TGFBR1, HDAC2, SHC2, TGFB2, GADD45G, AKT3, ABL1, RUNX1, PIK3R2, PIK3R1, HGF, TGFB3, KRAS* |
| Prolactin signaling pathway | 0.200 | 8.25E-04 | *SOCS5, SRC, SOCS3, SHC2, SOCS2, CCND2, GALT, AKT3, CISH, PIK3R1, HGF, MAPK8, KRAS, PIK3R2* |
| Calcium signaling pathway | 0.140 | 8.64E-04 | *PTGFR, PDGFRA, PLCG1, VDAC3, CYSLTR2, P2RX6, PDE1A, CAMK4, CACNA1G, ORAI2, TBXA2R, PRKCB, CAMK2D, OXTR, SLC25A6, PHKA2, HTR6, HTR4, PLCD3, TPCN1, ADCY2, PTK2B, CALML4, GNA11, PLN, AGTR1, SPR* |
| RNA transport | 0.145 | 1.01E-03 | *CYFIP2, PABPC1, EIF2B4, NUP85, EIF3H, EIF3I, EIF3E, NDC1, NUP188, EEF1A1, NUP210, SEC13, EEF1A2, XPOT, NUP107, NUP160, NUP93, ALYREF, THOC7, NUP210L, NUP54, NUP37, NUP155, NUP205* |
| Hypertrophic cardiomyopathy (HCM) | 0.178 | 1.11E-03 | *ITGA9, LAMA2, TNNT2, DMD, ACE, ITGB3, PRKAA1, CACNB4, TGFB2, ITGA11, ITGAV, IGF1, ACTB, CACNA2D1, TGFB3, CACNA2D4* |
| GnRH signaling pathway | 0.172 | 1.50E-03 | *SRC, PRKCB, PLD1, ADCY2, CAMK2D, MAP3K3, EGR1, JUN, CALML4, PTK2B, GNA11, HGF, PLA2G4A, MMP2, MAPK8, KRAS* |
| Arrhythmogenic right ventricular cardiomyopathy (ARVC) | 0.182 | 1.83E-03 | *ITGA9, LAMA2, CDH2, DMD, TCF7L1, TCF7L2, CACNB4, ITGB3, ITGA11, ITGAV, ACTB, CACNA2D1, LEF1, CACNA2D4* |
| Dilated cardiomyopathy (DCM) | 0.167 | 2.00E-03 | *ITGA9, ITGB3, TNNT2, DMD, ADCY2, LAMA2, ITGAV, CACNB4, TGFB2, ITGA11, IGF1, PLN, ACTB, CACNA2D1, TGFB3, CACNA2D4* |
| Fanconi anemia pathway | 0.204 | 2.54E-03 | *SLX4, FAAP100, WDR48, MLH1, APITD1, FANCG, FANCD2, RPA2, PMS2, FANCL, TOP3B* |
| MicroRNAs in cancer | 0.117 | 2.80E-03 | *HDAC1, RECK, MMP16, FOXP1, STMN1, TRIM71, ZFPM2, TGFB2, SPRY2, PLCG1, ABL1, ZEB1, DNMT1, TNXB, THBS1, PIK3R2, PDCD4, KRAS, PDGFA, ITGB3, PRKCB, TNC, HGF, RHOA, MDM4, PDGFRA, ZEB2, RPS6KA5, CASP3, CCND2, KIF23, VIM, MET, BMPR2, BCL2* |
| Platinum drug resistance | 0.178 | 3.07E-03 | *PMAIP1, CASP3, XPA, MLH1, AKT3, SLC31A1, MGST1, PIK3R2, PIK3R1, BCL2, ATP7A, BID, TOP2A* |
| Acute myeloid leukemia | 0.182 | 3.77E-03 | *SPI1, CSF1R, TCF7L1, TCF7L2, AKT3, PML, RUNX1, PIK3R2, PIK3R1, HGF, LEF1, KRAS* |
| Endocrine resistance | 0.153 | 5.49E-03 | *SRC, IGF1, ADCY2, SHC2, JUN, CARM1, HGF, AKT3, PIK3R2, PIK3R1, JAG2, MAPK8, KRAS, MMP2, BCL2* |
| Longevity regulating pathway | 0.157 | 5.80E-03 | *EHMT1, IGF1, ADCY2, SESN3, CREB5, CAMK4, PRKAA1, AKT3, APPL1, CREB3L1, PIK3R2, PIK3R1, TSC2, KRAS* |
| Neuroactive ligand-receptor interaction | 0.109 | 5.85E-03 | *PTGFR, P2RY13, UTS2R, CYSLTR2, OPRL1, GABRA3, P2RX6, CNR1, PTGER4, S1PR2, RXFP3, PTGER2, ADRA2C, GRIN3A, VIPR2, HRH3, VIPR1, EDN3, EDN2, CALCR, TBXA2R, GLP1R, LEPR, HTR6, HTR4, P2RY6, NTS, RLF, HTR1A, CHRNG, CHRND, OXTR, LPAR2, AGTR2, AGTR1, LPAR1, SPR* |
| Oxytocin signaling pathway | 0.131 | 7.35E-03 | *SRC, NPR2, PPP1CC, ADCY2, NPR1, CAMK2D, RHOA, PRKCB, JUN, CAMK4, PRKAA1, CACNB4, MAP2K5, OXTR, PLA2G4A, CACNA2D1, KRAS, ACTB, CACNA2D4, CALML4* |
| Glycine, serine and threonine metabolism | 0.200 | 1.04E-02 | *GAMT, GCAT, PSAT1, GATM, SARDH, DMGDH, ALDH7A1, CHDH* |
| Vitamin B6 metabolism | 0.500 | 1.63E-02 | *PSAT1, PDXP, PDXK* |
| cGMP-PKG signaling pathway | 0.120 | 1.64E-02 | *NPR1, NPR2, PPP1CC, ADCY2, CREB5, CNP, GTF2IRD1, SLC25A6, ADRA2C, AKT3, KCNMA1, CREB3L1, TRPC6, GNA11, VDAC3, PLN, AGTR1, GTF2I, RHOA, CALML4* |
| Alanine, aspartate and glutamate metabolism | 0.194 | 1.81E-02 | *GPT2, RIMKLB, ABAT, GFPT2, ASL, ASRGL1, CAD* |
| Amphetamine addiction | 0.147 | 2.62E-02 | *HDAC1, PPP1CC, CAMK2D, CREB5, PRKCB, JUN, CAMK4, CALML4, GRIN3A, CREB3L1* |
| Vascular smooth muscle contraction | 0.121 | 2.71E-02 | *NPR1, NPR2, ADCY2, ACTG2, RHOA, PRKCB, RAMP1, KCNMA1, CALML4, GNA11, CNP, AGTR1, PLA2G4A, EDN2, EDN3, PPP1CC* |
| RNA degradation | 0.139 | 2.79E-02 | *DCPS, DCP2, DIS3, BTG1, PARN, MPHOSPH6, ENO1, EXOSC8, PABPC1, CNOT2, CNOT6* |
| Lysine degradation | 0.153 | 2.83E-02 | *EHMT1, AADAT, AASS, EZH1, ALDH9A1, PRDM2, ALDH7A1, NSD1, SUV420H1* |
| Renin secretion | 0.145 | 2.83E-02 | *NPR1, ACE, PTGER4, PDE1A, KCNMA1, PTGER2, CALML4, EDN3, EDN2, AGTR1* |
| Histidine metabolism | 0.217 | 3.02E-02 | *AMDHD1, ALDH1A3, ALDH9A1, CNDP2, ALDH7A1* |
| Cholinergic synapse | 0.125 | 3.02E-02 | *ADCY2, CAMK2D, CREB5, PRKCB, FYN, CAMK4, GNG12, AKT3, CREB3L1, PIK3R2, PIK3R1, GNA11, KRAS, BCL2* |
| mRNA surveillance pathway | 0.132 | 3.11E-02 | *SMG6, PPP1CC, PABPC1, PPP2CB, ALYREF, PPP2R5D, CPSF6, NUDT21, PAPOLG, CPSF3, CPSF2, PPP2R5C* |
| Ubiquitin mediated proteolysis | 0.117 | 3.54E-02 | *SMURF1, FBXW7, NHLRC1, SMURF2, UBE2J1, SOCS3, UBE2Z, UBE2D1, PIAS2, UBA7, SAE1, PIAS1, PML, FANCL, ANAPC4, CDC20* |
| beta-Alanine metabolism | 0.182 | 3.57E-02 | *DPYD, ALDH9A1, ABAT, ALDH7A1, CNDP2, ALDH1A3* |
| Longevity regulating pathway-multiple species | 0.145 | 3.61E-02 | *HDAC1, HDAC2, ADCY2, PRKAA1, AKT3, PIK3R2, PIK3R1, KRAS, IGF1* |
| SNARE interactions in vesicular transport | 0.176 | 3.98E-02 | *SEC22B, SNAP23, USE1, GOSR2, GOSR1, BNIP1* |
| Vasopressin-regulated water reabsorption | 0.159 | 4.21E-02 | *AQP4, DLC1, DCTN6, CREB5, CREB3L1, ARHGDIB, DYNC2H1* |
| Prion diseases | 0.171 | 4.42E-02 | *GSS, EGR1, FYN, LAMC1, C7, NCAM1* |
