# Supplementary material for: Comparative transcriptomic analysis revealed potential mechanisms regulating the hypertrophy of goose pectoral muscles
Source: Poult Sci. 2024 Nov 2;103(12):104498. doi: 10.1016/j.psj.2024.104498 (PMC11577216; doi:10.1016/j.psj.2024.104498)
Supplement: Supplementary file 5 [file mmc5.docx]

***Supplementary Table S3. Functional enrichment analysis of DEGs that were identified from pectoral muscles between LD and SW geese at 6, 10, or 30 weeks of age.***

| *P* value |  | Rich factor |  |  |  | DEGs^2^ |
| --- | --- | --- | --- | --- | --- | --- |
|  |  |  |  | KEGG pathway^1^ |  |  |
|  |  |  |  | **6 W** |  |  |
| **Lipid metabolism** | | | | | | |
| 1.41E-05 |  | 0.081 |  | Adrenergic signaling in cardiomyocytes |  | *ADCY1, ADCY3, PPP2R2D, MAPK13, TPM2, ADRB1, RAPGEF4, CREB3L3, CREM, RAPGEF3, MYL3, TNNC1* |
| 6.55E-05 |  | 0.127 |  | Regulation of lipolysis in adipocytes |  | *ADCY1, ADRB1, ADCY3, PNPLA2, ADRB3, PRKG1, ABHD5* |
| 3.94E-03 |  | 0.070 |  | Insulin secretion |  | *ADCY1, ADCY3, RAPGEF4, CREB3L3, KCNU1, ADCYAP1R1* |
| 4.13E-03 |  | 0.054 |  | Phospholipase D signaling pathway |  | *AVPR1B, ADCY1, SHC3, ADCY3, KIT, RAPGEF4, RAPGEF3, KITLG* |
| 4.83E-03 |  | 0.058 |  | AMPK signaling pathway |  | *ULK1, PPP2R2D, PFKFB3, PPARGC1A, SCD5, CREB3L3, GYS2* |
| 5.66E-03 |  | 0.077 |  | Cortisol synthesis and secretion |  | *CACNA1H, CREB3L3, NR0B1, ADCY1, ADCY3* |
| 8.43E-03 |  | 0.069 |  | Bile secretion |  | *SLC4A4, SLC51B, ABCB4, ADCY1, ADCY3* |
| 9.43E-03 |  | 0.085 |  | Sphingolipid metabolism |  | *B4GALT6, SGMS2, ASAH2, SGMS1* |
| 1.66E-02 |  | 0.050 |  | Sphingolipid signaling pathway |  | *ASAH2, PPP2R2D, S1PR1, SGMS2, SGMS1, MAPK13* |
| 1.73E-02 |  | 0.039 |  | Thermogenesis |  | *NDUFA2, ADCY1, PNPLA2, ADCY3, PPARGC1A, CREB3L3, ADRB3, PRKG1, MAPK13* |
| 1.76E-02 |  | 0.097 |  | Mucin type O-glycan biosynthesis |  | *GCNT1, GALNT9, GALNT3* |
| 2.04E-02 |  | 0.067 |  | Steroid hormone biosynthesis |  | *SRD5A3, AKR1D1, UGT2B17, HSD11B2* |
| 2.14E-02 |  | 0.066 |  | Glycerolipid metabolism |  | *PNPLA2, DGAT2, PPAPDC1A, ALDH2* |
| 2.66E-02 |  | 0.051 |  | Aldosterone synthesis and secretion |  | *CACNA1H, CREB3L3, ADCY1, KCNJ5, ADCY3* |
| 3.05E-02 |  | 0.133 |  | Glycosphingolipid biosynthesis-ganglio series |  | *B3GALT4, ST6GALNAC3* |
| 3.51E-02 |  | 0.047 |  | Glucagon signaling pathway |  | *CREB3L3, GYS2, SIK1, PPARGC1A, PHKG1* |
| 3.51E-02 |  | 0.047 |  | Parathyroid hormone synthesis, secretion and action |  | *CREB3L3, PDE4B, PTHLH, ADCY1, ADCY3* |
| 3.84E-02 |  | 0.054 |  | Thyroid hormone synthesis |  | *CREB3L3, SLC26A4, ADCY1, ADCY3* |
| 4.84E-02 |  | 0.039 |  | Cushing syndrome |  | *WNT2B, CACNA1H, ADCY1, ADCY3, WNT3, CREB3L3* |
| **Proliferation/apoptosis** | | | | | | |
| 2.07E-05 |  | 0.067 |  | Rap1 signaling pathway |  | *RAPGEF4, ADCY1, ADCY3, ENAH, RAPGEF3, THBS1, GRIN2B, VEGFA, MAP2K6, MAGI1, MAPK13, KITLG, KIT, KDR* |
| 2.09E-05 |  | 0.085 |  | Purine metabolism |  | *ENTPD3, ADCY1, ADCY3, AMPD3, IMPDH2, AK4, PDE9A, GUCY1A2, ADA, POMP, PDE4B* |
| 4.22E-04 |  | 0.045 |  | PI3K-Akt signaling pathway |  | *IL2RB, IL4R, PPP2R2D, GNB3, THBS1, TLR2, CREB3L3, GYS2, FASLG, MAGI1, VEGFA, KITLG, KIT, PAK1, KDR, LAMA5* |
| 6.02E-04 |  | 0.047 |  | MAPK signaling pathway |  | *CACNA1H, MECOM, GADD45B, KIT, MAP3K12, FASLG, MAP2K6, MAPK13, DUSP5, VEGFA, KITLG, FLNC, KDR, MAPK8IP3* |
| 7.95E-04 |  | 0.125 |  | Ferroptosis |  | *MAP1LC3A, TF, MAP1LC3B, HMOX1, TFRC* |
| 1.05E-03 |  | 0.092 |  | Mitophagy-animal |  | *RAB7A, ULK1, PARK2, UBB, PINK1, BNIP3* |
| 2.13E-03 |  | 0.061 |  | FoxO signaling pathway |  | *GADD45B, PLK2, FASLG, FBXO32, MAPK13, BCL6, S1PR1, BNIP3* |
| 6.59E-03 |  | 0.043 |  | Ras signaling pathway |  | *SHC3, GNB3, KIT, GRIN2B, FASLG, ZAP70, VEGFA, KITLG, RASAL3, KDR* |
| 6.87E-03 |  | 0.049 |  | Jak-STAT signaling pathway |  | *IL2RB, IL20RB, IL4R, GFAP, IL23R, IL11RA, IL15RA, IL10RA* |
| 8.43E-03 |  | 0.069 |  | p53 signaling pathway |  | *AIFM2, CDS1, GADD45B, SESN1, THBS1* |
| 1.65E-02 |  | 0.058 |  | ECM-receptor interaction |  | *HMMR, CD44, SDC4, LAMA5, THBS1* |
| 2.89E-02 |  | 0.044 |  | Apoptosis |  | *CTSL, GADD45B, ENDOG, PARP3, FASLG, TUBA4A* |
| 3.87E-02 |  | 0.046 |  | HIF-1 signaling pathway |  | *TF, VEGFA, PFKFB3, TFRC, HMOX1* |
| 1.68E-05 |  | 0.045 |  | Pathways in cancer |  | *BIRC7, MGST3, IL23R, LAMA5, MECOM, GADD45B, CXCR4, WNT2B, IL4R, CKS2, GNB3, FASLG, KITLG, ARNT2, EPAS1, IL2RB, VEGFA, ADCY1, ADCY3, WNT3, HMOX1, KIT, DLL4, IL15RA* |
| 1.95E-03 |  | 0.062 |  | Relaxin signaling pathway |  | *ADCY1, SHC3, RXFP1, ADCY3, GNB3, CREB3L3, VEGFA, MAPK13* |
| 3.94E-02 |  | 0.041 |  | Breast cancer |  | *WNT2B, SHC3, WNT3, GADD45B, KIT, DLL4* |
| **Immune response** | | | | | | |
| 1.87E-04 |  | 0.051 |  | Cytokine-cytokine receptor interaction |  | *IL2RB, IL20RB, IL10RA, ACVR1C, PLEKHG5, IL23R, CD4, FASLG, CXCR6, CXCR4, IL11RA, CCL20, IL15RA, CXCL14, IL4R* |
| 2.11E-04 |  | 0.060 |  | Epstein-Barr virus infection |  | *ENTPD3, CD44, GADD45B, PSMD6, PSMD11, PSMD3, PSMD2, TLR2, CD247, MAP2K6, MAPK13, PSMD1* |
| 3.25E-04 |  | 0.082 |  | Hematopoietic cell lineage |  | *IL4R, CD44, KIT, CD4, IL11RA, TFRC, CD8A, KITLG* |
| 7.93E-04 |  | 0.071 |  | Leukocyte transendothelial migration |  | *CLDN10, CXCR4, CLDN14, RAPGEF3, MYL10, EZR, RAPGEF4, MAPK13* |
| 9.30E-04 |  | 0.080 |  | Gap junction |  | *ADCY1, ADCY3, ADRB1, GUCY1A2, PRKG1, HTR2B, TUBA4A* |
| 9.89E-04 |  | 0.079 |  | PD-L1 expression and PD-1 checkpoint pathway in cancer |  | *TLR2, TICAM1, CD4, CD247, MAP2K6, MAPK13, ZAP70* |
| 1.18E-03 |  | 0.076 |  | Th1 and Th2 cell differentiation |  | *IL2RB, IL4R, CD4, CD247, DLL4, ZAP70, MAPK13* |
| 1.86E-03 |  | 0.070 |  | Inflammatory mediator regulation of TRP channels |  | *ADCY1, ADCY3, HTR2B, TRPM8, MAP2K6, MAPK13, TRPA1* |
| 1.96E-03 |  | 0.069 |  | Melanogenesis |  | *WNT2B, ADCY1, ADCY3, WNT3, KIT, CREB3L3, KITLG* |
| 2.18E-03 |  | 0.068 |  | Chagas disease (American trypanosomiasis) |  | *TICAM1, ADCY1, PPP2R2D, TLR2, CD247, FASLG, MAPK13* |
| 2.66E-03 |  | 0.065 |  | Th17 cell differentiation |  | *IL2RB, IL4R, CD4, CD247, IL23R, MAPK13, ZAP70* |
| 4.34E-03 |  | 0.108 |  | Primary immunodeficiency |  | *CD4, CD8A, ZAP70, ADA* |
| 5.40E-03 |  | 0.044 |  | Human cytomegalovirus infection |  | *IL10RA, ADCY3, CXCR4, GNB3, CREB3L3, FASLG, MAP2K6, MAPK13, VEGFA, ADCY1* |
| 7.79E-03 |  | 0.060 |  | Viral protein interaction with cytokine and cytokine receptor |  | *IL2RB, IL20RB, IL10RA, CXCR4, CCL20, CXCL14* |
| 1.28E-02 |  | 0.048 |  | Cell adhesion molecules (CAMs) |  | *CLDN10, CLDN14, ALCAM, SDC4, CD4, CD226, CD8A* |
| 1.33E-02 |  | 0.053 |  | Toxoplasmosis |  | *LAMA5, BIRC7, TLR2, MAP2K6, MAPK13, IL10RA* |
| 1.45E-02 |  | 0.060 |  | Salmonella infection |  | *PYCARD, RAB7A, FLNC, PAK1, MAPK13* |
| 1.55E-02 |  | 0.073 |  | Legionellosis |  | *RAB1A, PYCARD, TLR2, BNIP3* |
| 1.56E-02 |  | 0.046 |  | Phagosome |  | *TLR2, RAB7A, CTSL, THBS1, ATP6V0A4, TFRC, TUBA4A* |
| 1.62E-02 |  | 0.042 |  | Chemokine signaling pathway |  | *ADCY1, SHC3, ADCY3, GNB3, CXCR6, CXCR4, CCL20, CXCL14* |
| 1.78E-02 |  | 0.050 |  | Yersinia infection |  | *TICAM1, ZAP70, MAPK13, PYCARD, PAK1, MAP2K6* |
| 2.03E-02 |  | 0.055 |  | Rheumatoid arthritis |  | *ATP6V0A4, CCL20, VEGFA, CTSL, TLR2* |
| 2.38E-02 |  | 0.053 |  | Amoebiasis |  | *TLR2, C8A, RAB7A, LAMA5, ADCY1* |
| 2.48E-02 |  | 0.033 |  | Human papillomavirus infection |  | *WNT2B, TICAM1, LAMA5, WNT3, PPP2R2D, THBS1, ATP6V0A4, CREB3L3, FASLG, MAGI1, VEGFA* |
| 2.48E-02 |  | 0.046 |  | Natural killer cell mediated cytotoxicity |  | *SHC3, SH2D1B, SH2D1A, CD247, FASLG, ZAP70* |
| 2.62E-02 |  | 0.041 |  | Tight junction |  | *CLDN10, CLDN14, PPP2R2D, EZR, MPP4, MAGI1, TUBA4A* |
| 2.81E-02 |  | 0.038 |  | Human immunodeficiency virus 1 infection |  | *TLR2, CXCR4, GNB3, CD4, CD247, FASLG, MAP2K6, MAPK13* |
| 3.17E-02 |  | 0.049 |  | T cell receptor signaling pathway |  | *CD4, CD8A, CD247, ZAP70, MAPK13* |
| 4.25E-02 |  | 0.045 |  | TNF signaling pathway |  | *CCL20, CREB3L3, MAP2K6, BAG4, MAPK13* |
| **Others** | | | | | | |
| 1.11E-13 |  | 0.047 |  | Metabolic pathways |  | *ST6GAL2, ACSBG2, AMPD3, AKR1D1, AGMAT, NDUFA2, MGST3, PDE9A, PIGC, SGMS2, SGMS1, GADL1, POMP, AK4, CHKA, TAT, MTHFD2L, GCNT1, ASAH2, GATM, AASS, DGAT2, AFMID, IMPDH2, PNPLA2, MPST, UGT2B17, MGAT5B, BCO1, ACOT12, HSD11B2, HNMT, B4GALT6, B3GALT4, DHFR, TDO2, ENTPD3, CRYL1, SCD5, NUDT12, GANC, PLCD4, GYS2, DDO, GALNT9, ADA, ST6GALNAC3, ACMSD, GALNT3, IVD, ALDH8A1, CMBL, SDSL, ADCY1, ADCY3, TSTA3, PFKFB3, HMOX1, GLUL, ALDH2, CA4, ATP6V0A4, OXCT1, PAH, GUCY1A2, PDE4B, CCBL1* |
| 1.59E-06 |  | 0.113 |  | Circadian entrainment |  | *CACNA1H, PER3, ADCY1, KCNJ5, ADCY3, GRIA1, GNB3, GRIN2B, GUCY1A2, PRKG1, ADCYAP1R1* |
| 2.16E-06 |  | 0.178 |  | Proteasome |  | *PSMD1, PSMD6, PSMD11, PSMD3, PSMD2, PSMA3, PSMA7, PSMB3* |
| 3.32E-06 |  | 0.074 |  | Proteoglycans in cancer |  | *WNT2B, CTSL, WNT3, CD44, SDC4, EZR, THBS1, TLR2, VEGFA, FASLG, HOXD10, MAPK13, HSPB2, FLNC, KDR* |
| 7.67E-06 |  | 0.056 |  | Neuroactive ligand-receptor interaction |  | *AVPR1B, CCKBR, ADRB1, RXFP1, GABRR1, S1PR1, GRIA1, GRIK2, CHRNA3, GRIN2B, OXT, SSTR3, ADRB3, HTR2B, GABRG2, HTR1F, ADCYAP1R1, GPR50, CHRNB3* |
| 9.87E-05 |  | 0.061 |  | cAMP signaling pathway |  | *RAPGEF4, ADCY1, ADCY3, OXT, GRIA1, ADRB1, GRIN2B, CREB3L3, TNNC1, RAPGEF3, PDE4B, ADCYAP1R1, HTR1F* |
| 1.23E-04 |  | 0.143 |  | Tryptophan metabolism |  | *TDO2, AFMID, ALDH2, ALDH8A1, CCBL1, ACMSD* |
| 1.89E-04 |  | 0.090 |  | GABAergic synapse |  | *SLC6A1, ADCY1, ADCY3, GABRG2, GNB3, SLC12A5, GLUL, GABRR1* |
| 2.78E-04 |  | 0.068 |  | Retrograde endocannabinoid signaling |  | *GRIA1, NDUFA2, ADCY1, KCNJ5, ADCY3, GABRG2, MAPK13, NAPEPLD, GNB3, GABRR1* |
| 5.52E-04 |  | 0.057 |  | Calcium signaling pathway |  | *AVPR1B, CCKBR, ADCY1, ADCY3, CXCR4, ADRB1, PLCD4, ADRB3, CACNA1H, HTR2B, PHKG1* |
| 6.80E-04 |  | 0.060 |  | cGMP-PKG signaling pathway |  | *ADCY1, ADCY3, ADRB3, ADRB1, CREB3L3, KCNU1, TRPC6, GUCY1A2, RGS2, PRKG1* |
| 7.40E-04 |  | 0.065 |  | Fluid shear stress and atherosclerosis |  | *TXN, CTSL, SDC4, HMOX1, MGST3, VEGFA, MAP2K6, MAPK13, KDR* |
| 9.89E-04 |  | 0.079 |  | Longevity regulating pathway |  | *ULK1, SESN1, ADCY3, PPARGC1A, CREB3L3, ATG13, ADCY1* |
| 1.08E-03 |  | 0.049 |  | Endocytosis |  | *IL2RB, CHMP4C, RAB11FIP4, RAB11FIP1, DNAJC6, KIF5C, RAB7A, PSD2, TFRC, UBB, CXCR4, DAB2* |
| 1.12E-03 |  | 0.077 |  | Morphine addiction |  | *ADCY1, KCNJ5, ADCY3, GABRG2, GNB3, GABRR1, PDE4B* |
| 2.04E-03 |  | 0.061 |  | Dopaminergic synapse |  | *KIF5C, KCNJ5, PPP2R2D, GRIA1, GNB3, GRIN2B, CREB3L3, MAPK13* |
| 3.71E-03 |  | 0.061 |  | Glutamatergic synapse |  | *ADCY1, ADCY3, GRIK2, GRIA1, GNB3, GRIN2B, GLUL* |
| 4.85E-03 |  | 0.067 |  | Salivary secretion |  | *ADCY1, ADCY3, ADRB1, ADRB3, PRKG1, GUCY1A2* |
| 5.24E-03 |  | 0.400 |  | Phenylalanine, tyrosine and tryptophan biosynthesis |  | *PAH, TAT* |
| 5.60E-03 |  | 0.100 |  | Nicotine addiction |  | *GRIN2B, GABRR1, GRIA1, GABRG2* |
| 6.49E-03 |  | 0.063 |  | Dilated cardiomyopathy (DCM) |  | *ADCY1, ADCY3, TPM2, ADRB1, MYL3, TNNC1* |
| 7.82E-03 |  | 0.053 |  | Vascular smooth muscle contraction |  | *AVPR1B, ADCY1, ADCY3, RAMP3, KCNU1, PRKG1, GUCY1A2* |
| 9.34E-03 |  | 0.125 |  | Vitamin digestion and absorption |  | *SLC52A3, MMACHC, SLC19A2* |
| 9.86E-03 |  | 0.067 |  | Gastric acid secretion |  | *CCKBR, ADCY1, EZR, KCNK10, ADCY3* |
| 1.08E-02 |  | 0.082 |  | Cysteine and methionine metabolism |  | *TAT, CCBL1, MPST, SDSL* |
| 1.14E-02 |  | 0.115 |  | Folate biosynthesis |  | *DHFR, PAH, GGH* |
| 1.22E-02 |  | 0.078 |  | Amyotrophic lateral sclerosis (ALS) |  | *GRIN2B, GRIA1, MAP2K6, MAPK13* |
| 1.61E-02 |  | 0.046 |  | Oxytocin signaling pathway |  | *ADCY1, KCNJ5, ADCY3, OXT, GUCY1A2, RGS2, TRPM2* |
| 2.66E-02 |  | 0.051 |  | Endocrine resistance |  | *MAPK13, SHC3, DLL4, ADCY1, ADCY3* |
| 2.85E-02 |  | 0.060 |  | Long-term potentiation |  | *GRIN2B, GRIA1, ADCY1, RAPGEF3* |
| 2.98E-02 |  | 0.059 |  | Amphetamine addiction |  | *GRIN2B, CREB3L3, GRIA1, ARC* |
| 3.12E-02 |  | 0.058 |  | Renin secretion |  | *ADRB1, ADRB3, ADCYAP1R1, GUCY1A2* |
| 3.75E-02 |  | 0.118 |  | Phenylalanine metabolism |  | *PAH, TAT* |
| 3.75E-02 |  | 0.118 |  | Nitrogen metabolism |  | *GLUL, CA4* |
| 3.91E-02 |  | 0.038 |  | Transcriptional misregulation in cancer |  | *WT1, IL2RB, GADD45B, ARNT2, BCL6, HPGD, HOXA9* |
| 3.99E-02 |  | 0.053 |  | Biosynthesis of amino acids |  | *PAH, TAT, GLUL, SDSL* |
| 4.25E-02 |  | 0.045 |  | Cholinergic synapse |  | *CREB3L3, CHRNA3, ADCY1, GNB3, ADCY3* |
| 4.65E-02 |  | 0.043 |  | Serotonergic synapse |  | *HTR1F, RAPGEF3, KCNJ5, GNB3, HTR2B* |
| 4.91E-02 |  | 0.100 |  | One carbon pool by folate |  | *DHFR, MTHFD2L* |
|  |  |  |  | **10 W** |  |  |
| **Lipid metabolism** | | | | | | |
| 3.11E-05 |  | 0.131 |  | Glycerolipid metabolism |  | *LIPG, GLYCTK, GPAT2, PNPLA2, MGAT2, AGPAT4, AGPAT2, MOGAT1* |
| 1.26E-03 |  | 0.174 |  | Mannose type O-glycan biosynthesis |  | *POMT1, B3GAT2, B3GAT1, MGAT5B* |
| 2.63E-03 |  | 0.072 |  | Glycerophospholipid metabolism |  | *GPAT2, ETNPPL, AGPAT4, AGPAT2, PLB1, PLA2G4F, GPCPD1* |
| 3.02E-03 |  | 0.133 |  | Pentose phosphate pathway |  | *GLYCTK, RGN, IDNK, PFKP* |
| 4.29E-03 |  | 0.091 |  | Regulation of lipolysis in adipocytes |  | *PTGER3, ABHD5, ADRB3, PNPLA2, PLIN1* |
| 1.56E-02 |  | 0.080 |  | Cholesterol metabolism |  | *ABCB11, NPC1, MRAP, LIPG* |
| 2.23E-02 |  | 0.097 |  | Mucin type O-glycan biosynthesis |  | *GCNT4, ST3GAL2, GALNT3* |
| 2.59E-02 |  | 0.091 |  | Fructose and mannose metabolism |  | *TSTA3, PFKFB3, PFKP* |
| 3.41E-02 |  | 0.081 |  | Thyroid cancer |  | *GADD45B, RXRG, LEF1* |
| 3.60E-02 |  | 0.133 |  | Glycosphingolipid biosynthesis-ganglio series |  | *ST3GAL2, ST6GALNAC3* |
| 4.34E-02 |  | 0.073 |  | Fat digestion and absorption |  | *MGAT2, AGPAT2, NPC1L1* |
| 4.68E-02 |  | 0.056 |  | Bile secretion |  | *SLC51A, ABCB11, ABCB4, AQP9* |
| **Proliferation/apoptosis** | | | | | | |
| 5.12E-05 |  | 0.105 |  | ECM-receptor interaction |  | *HMMR, FRAS1, ITGB6, ITGA2B, TNR, SDC4, NPNT, FREM1, SV2B* |
| 8.97E-04 |  | 0.069 |  | Purine metabolism |  | *ENPP4, HPRT1, NT5C3B, PDE9A, PDE10A, ADSL, ENTPD8, PDE4D, PDE11A* |
| 3.43E-03 |  | 0.056 |  | Wnt signaling pathway |  | *RSPO3, NFATC1, RSPO1, SFRP4, LGR5, WNT6, SFRP2, PPARD, LEF1* |
| 6.75E-03 |  | 0.040 |  | PI3K-Akt signaling pathway |  | *BRCA1, TNR, ITGA2B, ITGB6, CHRM2, PPP2R2B, FOXO3, FGF7, GYS2, VEGFA, TLR4, LPAR2, FGFR3, PAK1* |
| 1.71E-02 |  | 0.055 |  | HIF-1 signaling pathway |  | *MKNK2, PFKFB3, HMOX1, PFKP, VEGFA, TLR4* |
| 1.72E-02 |  | 0.043 |  | Rap1 signaling pathway |  | *RAPGEF4, ITGA2B, FGF7, GRIN2B, RAPGEF5, PLCE1, LPAR2, VEGFA, FGFR3* |
| 3.47E-02 |  | 0.062 |  | Mitophagy-animal |  | *MITF, FOXO3, OPTN, PARK2* |
| 4.79E-02 |  | 0.034 |  | MAPK signaling pathway |  | *NFATC1, TGFB3, HSPB1, MKNK2, GADD45B, FGFR3, VEGFA, FGF7, DUSP4, PLA2G4F* |
| 2.68E-05 |  | 0.047 |  | Pathways in cancer |  | *TGFB3, NQO1, SMO, IL23R, MITF, STAT4, ZBTB16, ITGA2B, GADD45B, EML4, PTGER3, GLI2, GLI1, CXCR4, RXRG, PPARD, VEGFA, FGF7, LEF1, FGFR3, BDKRB2, HMOX1, WNT6, LPAR2, IL15RA* |
| 1.44E-03 |  | 0.095 |  | Basal cell carcinoma |  | *GLI2, GADD45B, WNT6, SMO, GLI1, LEF1* |
| 3.63E-02 |  | 0.061 |  | Non-small cell lung cancer |  | *FOXO3, GADD45B, RXRG, EML4* |
| **Immune response** | | | | | | |
| 1.67E-03 |  | 0.092 |  | Inflammatory bowel disease (IBD) |  | *NFATC1, TGFB3, STAT4, IL23R, TLR4, GATA3* |
| 1.88E-03 |  | 0.077 |  | Rheumatoid arthritis |  | *TGFB3, ATP6AP1, ATP6V0A4, CTSK, VEGFA, TLR4, ATP6V1B1* |
| 1.18E-02 |  | 0.060 |  | Inflammatory mediator regulation of TRP channels |  | *PRKCH, BDKRB2, PRKCQ, TRPV4, PLA2G4F, TRPV3* |
| 2.01E-02 |  | 0.048 |  | Cell adhesion molecules (CAMs) |  | *CLDN10, F3, NLGN1, SDC3, SDC4, NFASC, SIGLEC1* |
| 2.42E-02 |  | 0.046 |  | Phagosome |  | *MARCO, PLA2R1, TUBB4B, ATP6AP1, ATP6V0A4, TLR4, ATP6V1B1* |
| 3.98E-02 |  | 0.041 |  | Tight junction |  | *CLDN10, MYH7B, MYL12B, EPB41L4B, PPP2R2B, SYNPO, MPP4* |
| 4.31E-02 |  | 0.057 |  | Epithelial cell signaling in Helicobacter pylori infection |  | *ATP6V0A4, ATP6V1B1, ATP6AP1, PTPRZ1* |
| 4.96E-02 |  | 0.039 |  | Tuberculosis |  | *CD74, TGFB3, PLA2R1, ATP6AP1, ATP6V0A4, CARD9, TLR4* |
| **Others** | | | | | | |
| 5.24E-17 |  | 0.109 |  | Neuroactive ligand-receptor interaction |  | *PTGFR, GABRG2, P2RX5, GRIA1, GPR50, CRH, P2RY13, CYSLTR2, SSTR1, UTS2R, GABRA1, RXFP3, ADRA2A, PTGER3, GRIN3A, GRIN3B, GRIA3, PENK, GNRH1, CHRM2, NPFFR1, GABRE, P2RY8, CHRNA3, GCGR, MC5R, BDKRB2, CHRNA5, GRIK3, DRD1, GRIN2B, ADRB3, NPBWR1, OXTR, LPAR2, GABRD, HTR1F* |
| 1.94E-11 |  | 0.046 |  | Metabolic pathways |  | *PGAP1, GLYCTK, GPAT2, SHMT2, NQO1, PFKP, PAH, PDE9A, PLCE1, HAAO, GLUL, GADL1, B3GAT2, ADSL, ETNPPL, PDE11A, RGN, ALDH1A3, ADI1, NMNAT3, HPRT1, ATP6AP1, MPST, PLA2G4F, CHSY3, PNPLA2, FAH, MGAT5B, IDNK, MTMR7, ATP6V1B1, HSD17B2, HSD11B2, ST3GAL2, ALDH3B1, TDO2, LIPG, GCNT4, ENPP4, ENTPD8, NUDT12, NT5C3B, IMPA2, GYS2, B3GAT1, PLB1, PTGES, DHCR7, ST6GALNAC3, PDE10A, GALNT3, CMBL, PSAT1, MTHFD2, GANC, TSTA3, PFKFB3, HMOX1, ASNS, CA4, ATP6V0A4, AGPAT4, ELOVL2, AGPAT2, POMT1, PDE4D* |
| 1.85E-07 |  | 0.225 |  | Nicotine addiction |  | *GRIN2B, GRIA3, GRIA1, GRIN3A, GRIN3B, GABRG2, GABRE, GABRD, GABRA1* |
| 2.27E-07 |  | 0.084 |  | cAMP signaling pathway |  | *NFATC1, RYR2, GRIN2B, RAPGEF4, GRIA3, GLI1, GRIA1, CHRM2, DRD1, GRIN3A, SSTR1, GRIN3B, PLCE1, OXTR, HTR1F, PTGER3, PDE4D, PDE10A* |
| 7.67E-05 |  | 0.099 |  | Morphine addiction |  | *KCNJ6, GABRA1, GABRG2, DRD1, GABRE, GABRD, PDE11A, PDE4D, PDE10A* |
| 9.19E-05 |  | 0.067 |  | Calcium signaling pathway |  | *PTGFR, BDKRB2, CXCR4, CAMK1G, P2RX5, RYR2, CHRM2, PTGER3, ADRB3, PLCE1, OXTR, DRD1, CYSLTR2* |
| 3.48E-04 |  | 0.090 |  | GABAergic synapse |  | *SLC6A1, KCNJ6, GABRG2, SLC12A5, GLUL, GABRE, GABRD, GABRA1* |
| 7.05E-04 |  | 0.061 |  | Axon guidance |  | *PLXNB1, MYL12B, EPHA7, EPHA6, EPHA5, PLXNA4, SMO, TRPC6, SLIT1, CXCR4, ROBO3* |
| 8.03E-04 |  | 0.056 |  | Regulation of actin cytoskeleton |  | *BDKRB2, MYL12B, DIAPH3, ITGA2B, ITGB6, CHRM2, FGF7, INSRR, CXCR4, LPAR2, FGFR3, GSN* |
| 1.58E-03 |  | 0.070 |  | Glutamatergic synapse |  | *GRIN2B, GRIA1, GRIA3, GRIK3, GRIN3A, GLUL, GRIN3B, PLA2G4F* |
| 2.07E-03 |  | 0.088 |  | Amphetamine addiction |  | *GRIN2B, GRIA3, GRIA1, DRD1, GRIN3A, GRIN3B* |
| 3.26E-03 |  | 0.080 |  | Biosynthesis of amino acids |  | *ASNS, PFKP, PSAT1, GLUL, PAH, SHMT2* |
| 3.68E-03 |  | 0.078 |  | Arrhythmogenic right ventricular cardiomyopathy (ARVC) |  | *ITGB6, SGCG, ITGA2B, RYR2, DSP, LEF1* |
| 5.10E-03 |  | 0.176 |  | Phenylalanine metabolism |  | *PAH, ALDH3B1, ALDH1A3* |
| 7.46E-03 |  | 0.067 |  | Hypertrophic cardiomyopathy (HCM) |  | *TGFB3, ITGB6, SGCG, ITGA2B, RYR2, ACTC1* |
| 8.56E-03 |  | 0.048 |  | Transcriptional misregulation in cancer |  | *MLF1, ZBTB16, GRIA3, GADD45B, RXRG, FEV, PAX3, MITF, HPGD* |
| 9.92E-03 |  | 0.063 |  | Dilated cardiomyopathy (DCM) |  | *TGFB3, ITGB6, SGCG, ITGA2B, RYR2, ACTC1* |
| 1.08E-02 |  | 0.050 |  | Cellular senescence |  | *NFATC1, TGFB3, RAD9B, GADD45B, FOXO3, CDK1, FOXM1, TRPV4* |
| 1.46E-02 |  | 0.082 |  | Cocaine addiction |  | *GRIN3A, GRIN2B, GRIN3B, DRD1* |
| 1.66E-02 |  | 0.078 |  | Amyotrophic lateral sclerosis (ALS) |  | *GRIN2B, NEFL, GRIA1, TNFRSF1B* |
| 2.06E-02 |  | 0.100 |  | Nicotinate and nicotinamide metabolism |  | *NUDT12, NMNAT3, NT5C3B* |
| 2.06E-02 |  | 0.100 |  | Glyoxylate and dicarboxylate metabolism |  | *GLUL, GLYCTK, SHMT2* |
| 2.14E-02 |  | 0.047 |  | Retrograde endocannabinoid signaling |  | *KCNJ6, GRIA3, GRIA1, GABRG2, GABRE, GABRD, GABRA1* |
| 2.30E-02 |  | 0.051 |  | Carbon metabolism |  | *GLYCTK, PFKP, PSAT1, IDNK, SHMT2, RGN* |
| 2.59E-02 |  | 0.091 |  | beta-Alanine metabolism |  | *GADL1, ALDH3B1, ALDH1A3* |
| 2.73E-02 |  | 0.067 |  | Long-term depression |  | *GRIA3, CRH, GRIA1, PLA2G4F* |
| 2.82E-02 |  | 0.049 |  | Lysosome |  | *ARSG, GNPTAB, ATP6AP1, ATP6V0A4, NPC1, CTSK* |
| 3.19E-02 |  | 0.083 |  | Alanine, aspartate and glutamate metabolism |  | *ADSL, ASNS, GLUL* |
| 3.19E-02 |  | 0.083 |  | Tyrosine metabolism |  | *ALDH3B1, FAH, ALDH1A3* |
| 3.59E-02 |  | 0.052 |  | Circadian entrainment |  | *GRIN2B, GRIA3, GRIA1, RYR2, KCNJ6* |
| 3.63E-02 |  | 0.046 |  | Dopaminergic synapse |  | *KCNJ6, GRIA3, GRIA1, PPP2R2B, GRIN2B, DRD1* |
| 4.09E-02 |  | 0.075 |  | Glycine, serine and threonine metabolism |  | *PSAT1, GLYCTK, SHMT2* |
| 4.43E-02 |  | 0.118 |  | Nitrogen metabolism |  | *GLUL, CA4* |
| 4.57E-02 |  | 0.043 |  | Fluid shear stress and atherosclerosis |  | *HMOX1, ITGA2B, SDC4, NQO1, VEGFA, TRPV4* |
|  |  |  |  | **30 W** |  |  |
| **Lipid metabolism** | | | | | | |
| 3.79E-06 |  | 0.108 |  | Phospholipase D signaling pathway |  | *PDGFRA, ADCY2, F2R, PIP5K1B, PIK3CD, MRAS, EGF, RAPGEF4, PIK3R6, DGKH, AGPAT4, PIK3R3, AGPAT2, AGPAT3, PLA2G4F, GNA13* |
| 1.80E-05 |  | 0.124 |  | Glycerophospholipid metabolism |  | *PTDSS2, PCYT1B, LPIN3, CKB, GPAT2, ETNPPL, DGKH, AGPAT4, GPD1, AGPAT2, AGPAT3, PLA2G4F* |
| 3.12E-04 |  | 0.131 |  | Glycerolipid metabolism |  | *LPIN3, DGAT2, GPAT2, PNPLA2, DGKH, AGPAT4, AGPAT3, AGPAT2* |
| 6.03E-04 |  | 0.118 |  | Glycolysis/Gluconeogenesis |  | *ALDH3B1, GPI, PGM1, GAPDH, TPI1, ALDOC, PGK1, PKM* |
| 6.59E-04 |  | 0.116 |  | Adipocytokine signaling pathway |  | *PRKAG2, SOCS3, PRKAB1, RXRG, TRADD, LEPR, ACSBG1, ACSBG2* |
| 7.41E-04 |  | 0.081 |  | Adrenergic signaling in cardiomyocytes |  | *TPM4, ADCY2, CALM1, CAMK2D, ATP1B4, PPP2R3A, TPM2, PPP2R2B, RAPGEF4, PIK3R6, ATF4, SCN4B* |
| 7.51E-04 |  | 0.093 |  | Insulin resistance |  | *RPS6KA3, PPP1R3C, PPP1R3B, SOCS3, PRKAB1, PRKAG2, PIK3CD, PPP1R3D, PIK3R3, MLXIP* |
| 8.50E-04 |  | 0.111 |  | Bile secretion |  | *NCEH1, ADCY2, ATP1B4, SLC10A1, AQP4, AQP9, ABCB11, ABCC3* |
| 8.63E-04 |  | 0.127 |  | Regulation of lipolysis in adipocytes |  | *ADCY2, PIK3CD, PTGER3, PNPLA2, PIK3R3, TSHR, ABHD5* |
| 1.46E-03 |  | 0.136 |  | Carbohydrate digestion and absorption |  | *LCT, SLC37A4, ATP1B4, PIK3CD, PIK3R3, SLC2A5* |
| 3.74E-03 |  | 0.135 |  | Aldosterone-regulated sodium reabsorption |  | *PIK3CD, ATP1B4, PIK3R3, SGK1, SCNN1A* |
| 3.86E-03 |  | 0.073 |  | Insulin signaling pathway |  | *PPP1R3D, PPP1R3C, PPP1R3B, SOCS3, CALM1, PRKAB1, PIK3CD, PRKAG2, PRKAR2B, PIK3R3* |
| 4.05E-03 |  | 0.072 |  | Estrogen signaling pathway |  | *HSPA2, KCNJ6, ADCY2, CALM1, GNAO1, HSPA8, PIK3CD, PGR, ATF4, PIK3R3* |
| 6.67E-03 |  | 0.067 |  | Non-alcoholic fatty liver disease (NAFLD) |  | *SOCS3, NDUFB9, PRKAB1, PRKAG2, PIK3CD, LEPR, MLXIP, PIK3R3, NDUFS6, ATF4* |
| 7.75E-03 |  | 0.075 |  | Parathyroid hormone synthesis, secretion and action |  | *MMP16, SOST, ADCY2, RXRG, MMP17, GNA13, ATF4, FGFR1* |
| 8.56E-03 |  | 0.065 |  | Cushing syndrome |  | *CACNA1H, NCEH1, ADCY2, CCNE2, WNT6, KCNK2, WNT4, CAMK2D, ATF4, LEF1* |
| 9.47E-03 |  | 0.200 |  | Glycosphingolipid biosynthesis-ganglio series |  | *B3GALT4, ST6GALNAC3, ST8SIA1* |
| 9.75E-03 |  | 0.133 |  | Pentose phosphate pathway |  | *ALDOC, PGM1, DERA, GPI* |
| 1.46E-02 |  | 0.167 |  | Fatty acid biosynthesis |  | *ACSF3, ACSBG1, ACSBG2* |
| 1.48E-02 |  | 0.081 |  | Thyroid hormone synthesis |  | *IYD, ADCY2, ATP1B4, SLC26A4, ATF4, TSHR* |
| 1.49E-02 |  | 0.067 |  | AMPK signaling pathway |  | *CAB39L, PRKAG2, PRKAB1, PPP2R3A, PIK3CD, LEPR, PIK3R3, PPP2R2B* |
| 1.60E-02 |  | 0.071 |  | Aldosterone synthesis and secretion |  | *CACNA1H, ADCY2, CAMK1G, CALM1, CAMK2D, ATP1B4, ATF4* |
| 1.66E-02 |  | 0.158 |  | Pantothenate and CoA biosynthesis |  | *GADL1, PANK3, AASDHPPT* |
| 1.68E-02 |  | 0.071 |  | Progesterone-mediated oocyte maturation |  | *CCNB1, RPS6KA3, ADCY2, CPEB1, PIK3CD, PGR, PIK3R3* |
| 1.68E-02 |  | 0.071 |  | Phosphatidylinositol signaling system |  | *CALM1, PIP5K1B, PIK3CD, INPP4B, DGKH, PIK3R3, PLCD3* |
| 2.30E-02 |  | 0.066 |  | Glucagon signaling pathway |  | *ADCY2, CALM1, CAMK2D, PRKAG2, ATF4, PRKAB1, PKM* |
| 2.74E-02 |  | 0.070 |  | Insulin secretion |  | *ADCY2, CAMK2D, ATP1B4, RAPGEF4, ATF4, ADCYAP1R1* |
| 3.03E-02 |  | 0.077 |  | Cortisol synthesis and secretion |  | *CACNA1H, ATF4, NCEH1, ADCY2, KCNK2* |
| 3.33E-02 |  | 0.089 |  | ABC transporters |  | *ABCG1, ABCA12, ABCB11, ABCC3* |
| 3.55E-02 |  | 0.087 |  | Type II diabetes mellitus |  | *PIK3CD, PIK3R3, SOCS3, PKM* |
| 3.78E-02 |  | 0.085 |  | Ether lipid metabolism |  | *ENPP6, PLA2G7, ENPP2, PLA2G4F* |
| 3.84E-02 |  | 0.059 |  | Sphingolipid signaling pathway |  | *ASAH2, PPP2R3A, PIK3CD, TRADD, PPP2R2B, PIK3R3, GNA13* |
| 3.84E-02 |  | 0.059 |  | Thyroid hormone signaling pathway |  | *ATP1B4, RXRG, PIK3CD, WNT4, PIK3R3, PLCD3, DIO2* |
| 4.02E-02 |  | 0.182 |  | Taurine and hypotaurine metabolism |  | *GADL1, CDO1* |
| 4.02E-02 |  | 0.182 |  | Ubiquinone and other terpenoid-quinone biosynthesis |  | *TAT, NQO1* |
| 4.71E-02 |  | 0.068 |  | Inositol phosphate metabolism |  | *PIP5K1B, TPI1, PIK3CD, INPP4B, PLCD3* |
| 1.53E-03 |  | 0.091 |  | Choline metabolism in cancer |  | *PDGFRA, PCYT1B, CKB, PIP5K1B, PIK3CD, EGF, DGKH, PIK3R3, PLA2G4F* |
| **Proliferation/apoptosis** | | | | | | |
| 1.18E-05 |  | 0.071 |  | PI3K-Akt signaling pathway |  | *ITGA8, BRCA1, PDGFRA, PPP2R3A, EGF, PGF, CCNE2, PIK3CD, THBS3, GNG12, PIK3R6, FGF19, PIK3R3, MYB, FGF16, ITGB1, PPP2R2B, TNC, LAMC2, FGFR4, FGFR1, SGK1, F2R, TLR4, ATF4* |
| 1.87E-05 |  | 0.086 |  | Rap1 signaling pathway |  | *ITGB1, PGF, PDGFRA, ADCY2, CALM1, GNAO1, SKAP1, PIK3CD, MRAS, FGF16, RAPGEF4, F2R, FGF19, PIK3R3, MAP2K6, FGFR4, EGF, FGFR1* |
| 3.53E-05 |  | 0.094 |  | Wnt signaling pathway |  | *RSPO3, NFATC1, NFATC2, SOST, SFRP4, SFRP5, CAMK2D, DAAM1, INVS, DKK2, RSPO1, WNT4, GPC4, WNT6, LEF1* |
| 5.16E-05 |  | 0.071 |  | MAPK signaling pathway |  | *PGF, NFATC1, PDGFRA, RPS6KA3, PTPRR, HSPA2, TRADD, HSPA8, MRAS, MAP3K4, EGF, FGF19, CACNA1H, MAP2K6, ELK4, ATF4, FGF16, FGFR4, GNG12, PLA2G4F, FGFR1* |
| 6.12E-04 |  | 0.105 |  | ECM-receptor interaction |  | *ITGA8, HMMR, FRAS1, THBS3, AGRN, NPNT, ITGB1, TNC, LAMC2* |
| 9.19E-04 |  | 0.078 |  | mTOR signaling pathway |  | *CAB39L, RRAGD, RPS6KA3, SGK1, GRB10, PIK3CD, ATP6V1H, WDR59, WNT4, NPR3, PIK3R3, WNT6* |
| 9.38E-04 |  | 0.083 |  | FoxO signaling pathway |  | *CCNB1, TNFSF10, GABARAPL1, SGK1, PRKAB1, PRKAG2, PIK3CD, PIK3R3, HOMER3, BCL6, EGF* |
| 3.33E-03 |  | 0.080 |  | TNF signaling pathway |  | *TRADD, SOCS3, PIK3CD, BIRC2, TNFAIP3, CASP10, PIK3R3, MAP2K6, ATF4* |
| 3.68E-03 |  | 0.060 |  | Ras signaling pathway |  | *PGF, PDGFRA, FGF16, CALM1, PIK3CD, MRAS, GNG12, PLA2G4F, FGF19, PIK3R3, KSR1, FGFR4, EGF, FGFR1* |
| 8.22E-03 |  | 0.065 |  | Hippo signaling pathway |  | *SMAD7, BIRC2, PPP2R2B, CTGF, WNT4, WNT6, SAV1, LEF1, RASSF6, CTNNA3* |
| 8.48E-03 |  | 0.092 |  | Mitophagy-animal |  | *GABARAPL1, MRAS, PARK2, UBB, USP15, ATF4* |
| 9.01E-03 |  | 0.073 |  | HIF-1 signaling pathway |  | *CAMK2D, PIK3CD, GAPDH, PIK3R3, TLR4, ALDOC, EGF, PGK1* |
| 1.01E-02 |  | 0.104 |  | Amino sugar and nucleotide sugar metabolism |  | *TSTA3, GNPDA1, PGM1, UAP1, GPI* |
| 1.06E-02 |  | 0.066 |  | Apoptosis |  | *TNFSF10, ENDOG, PIK3CD, BIRC2, TRADD, PIDD1, CASP10, PIK3R3, ATF4* |
| 1.32E-02 |  | 0.074 |  | TGF-beta signaling pathway |  | *GREM2, THSD4, SMAD7, INHBA, FMOD, PITX2, TGIF1* |
| 1.71E-02 |  | 0.111 |  | DNA replication |  | *RFC4, MCM5, POLE, RFC2* |
| 2.07E-02 |  | 0.063 |  | Oocyte meiosis |  | *CCNB1, RPS6KA3, ADCY2, CALM1, CCNE2, CPEB1, CAMK2D, PGR* |
| 3.78E-02 |  | 0.085 |  | Nucleotide excision repair |  | *RFC4, XPA, POLE, RFC2* |
| 4.30E-02 |  | 0.069 |  | p53 signaling pathway |  | *CCNE2, AIFM2, CCNB1, PIDD1, CHEK1* |
| 3.74E-05 |  | 0.058 |  | Pathways in cancer |  | *PDGFRA, BIRC2, NQO1, EML4, LEF1, PGF, SPI1, CALM1, CCNE2, PIK3CD, GNG12, PTGER3, FGF19, PIK3R3, CTNNA3, EGF, DAPK2, ITGB1, CAMK2D, CKS2, RXRG, LAMC2, FGFR4, FGFR1, BDKRB1, FGF16, ADCY2, WNT6, WNT4, F2R, GNA13* |
| 2.09E-03 |  | 0.075 |  | Breast cancer |  | *BRCA1, FGF16, PIK3CD, WNT6, WNT4, FGF19, PGR, PIK3R3, LEF1, EGF, FGFR1* |
| 2.31E-03 |  | 0.074 |  | Gastric cancer |  | *FGF16, CCNE2, RXRG, PIK3CD, WNT6, WNT4, FGF19, PIK3R3, LEF1, EGF, CTNNA3* |
| 3.76E-03 |  | 0.086 |  | Small cell lung cancer |  | *ITGB1, CCNE2, CKS2, RXRG, PIK3CD, BIRC2, PIK3R3, LAMC2* |
| 1.94E-02 |  | 0.076 |  | EGFR tyrosine kinase inhibitor resistance |  | *PDGFRA, PIK3CD, PIK3R3, NRG1, EGF, GAS6* |
| 2.02E-02 |  | 0.086 |  | Endometrial cancer |  | *PIK3CD, LEF1, PIK3R3, EGF, CTNNA3* |
| 2.24E-02 |  | 0.062 |  | Relaxin signaling pathway |  | *ACTA2, ADCY2, GNAO1, RXFP3, PIK3CD, GNG12, PIK3R3, ATF4* |
| 3.19E-02 |  | 0.076 |  | Non-small cell lung cancer |  | *PIK3CD, RXRG, PIK3R3, EGF, EML4* |
| **Immune response** | | | | | | |
| 4.03E-05 |  | 0.105 |  | Platelet activation |  | *ITGB1, MYL12B, ADCY2, F2R, MYLK4, PIK3CD, PIK3R6, PIK3R3, PPP1R12A, P2RX1, PLA2G4F, BTK, GNA13* |
| 1.05E-04 |  | 0.110 |  | Inflammatory mediator regulation of TRP channels |  | *BDKRB1, PRKCH, ADCY2, CALM1, CAMK2D, PIK3CD, PIK3R3, MAP2K6, F2RL1, PLA2G4F, TRPV3* |
| 3.32E-04 |  | 0.075 |  | Focal adhesion |  | *ITGA8, ITGB1, PDGFRA, MYL12B, MYL10, MYLK4, PIK3CD, THBS3, BIRC2, PGF, PIK3R3, PPP1R12A, TNC, LAMC2, EGF* |
| 5.73E-04 |  | 0.096 |  | C-type lectin receptor signaling pathway |  | *IL17D, NFATC1, NFATC2, CALM1, PIK3CD, MRAS, CARD9, PIK3R3, KSR1, PYCARD* |
| 6.27E-04 |  | 0.082 |  | Cell adhesion molecules (CAMs) |  | *ITGA8, NEGR1, CNTNAP1, NTNG2, CLDN20, CD34, IGSF11, CD4, PVRL3, ITGB1, CD8A, CDH4* |
| 7.67E-04 |  | 0.101 |  | PD-L1 expression and PD-1 checkpoint pathway in cancer |  | *NFATC1, NFATC2, PIK3CD, EML4, CD4, PIK3R3, MAP2K6, TLR4, EGF* |
| 1.04E-03 |  | 0.088 |  | Toxoplasmosis |  | *ITGB1, HSPA2, IL10RA, GNAO1, HSPA8, BIRC2, PIK3R6, MAP2K6, TLR4, LAMC2* |
| 1.09E-03 |  | 0.067 |  | Human cytomegalovirus infection |  | *NFATC1, NFATC2, TRADD, IL10RA, ADCY2, CALM1, GNAO1, PIK3CD, PDGFRA, GNG12, PTGER3, PIK3R3, MAP2K6, ATF4, GNA13* |
| 1.11E-03 |  | 0.073 |  | Tuberculosis |  | *CASP10, IL10RA, CALM1, PLA2R1, CCK, ATP6V1H, TRADD, ATP6V0A4, CAMK2D, CARD9, TLR4, KSR1, ATP6V0D2* |
| 1.67E-03 |  | 0.083 |  | Yersinia infection |  | *ITGB1, NFATC1, NFATC2, RPS6KA3, PIP5K1B, PIK3CD, PIK3R3, MAP2K6, TLR4, PYCARD* |
| 3.05E-03 |  | 0.055 |  | Human papillomavirus infection |  | *ITGA8, ITGB1, CCNE2, PPP2R3A, PIK3CD, THBS3, TRADD, ATP6V1H, ATP6V0A4, WNT4, PIK3R3, TNC, WNT6, LAMC2, ATP6V0D2, PPP2R2B, EGF, PKM* |
| 3.54E-03 |  | 0.097 |  | Melanoma |  | *PDGFRA, PIK3CD, FGF16, FGF19, PIK3R3, EGF, FGFR1* |
| 4.20E-03 |  | 0.065 |  | Kaposi sarcoma-associated herpesvirus infection |  | *NFATC1, NFATC2, TRADD, GABARAPL1, CALM1, PIK3CD, GNG12, PIK3R6, PIK3R3, MAP2K6, UBB, HCK* |
| 4.43E-03 |  | 0.061 |  | Human immunodeficiency virus 1 infection |  | *NFATC1, NFATC2, TRADD, CALM1, GNAO1, PIK3CD, CCNB1, GNG12, CD4, PIK3R3, MAP2K6, TLR4, CHEK1* |
| 5.70E-03 |  | 0.059 |  | Human T-cell leukemia virus 1 infection |  | *NFATC1, NFATC2, SPI1, ADCY2, BUB1B, CCNE2, PIK3CD, CD4, PIK3R3, ELK4, ATF4, VAC14, CHEK1* |
| 1.04E-02 |  | 0.071 |  | Leukocyte transendothelial migration |  | *ITGB1, MYL12B, MYL10, CLDN20, PIK3CD, RAPGEF4, PIK3R3, CTNNA3* |
| 1.17E-02 |  | 0.061 |  | Hepatitis B |  | *NFATC1, NFATC2, CCNE2, PIK3CD, SLC10A1, CASP10, PIK3R3, MAP2K6, TLR4, ATF4* |
| 1.85E-02 |  | 0.069 |  | Melanogenesis |  | *ADCY2, CALM1, CAMK2D, GNAO1, WNT6, WNT4, LEF1* |
| 1.86E-02 |  | 0.108 |  | Primary immunodeficiency |  | *CD4, CD8A, BTK, ADA* |
| 3.00E-02 |  | 0.058 |  | Measles |  | *HSPA2, CCNE2, HSPA8, PIK3CD, TNFAIP3, PIK3R3, TRADD, TLR4* |
| 3.54E-02 |  | 0.074 |  | Fc epsilon RI signaling pathway |  | *PIK3CD, PIK3R3, MAP2K6, BTK, PLA2G4F* |
| 3.58E-02 |  | 0.053 |  | Tight junction |  | *ITGB1, MYH11, PRKAG2, MYL12B, HSPA4, CLDN20, PPP2R2B, WHAMM, PRKAB1* |
| 3.89E-02 |  | 0.064 |  | Fc gamma R-mediated phagocytosis |  | *PIP5K1B, PIK3CD, PIK3R3, HCK, PLA2G4F, MYO10* |
| 4.51E-02 |  | 0.051 |  | NOD-like receptor signaling pathway |  | *GABARAPL1, CCK, BIRC2, TNFAIP3, CARD9, TLR4, PYCARD, NLRX1, TRPM2* |
| 4.71E-02 |  | 0.068 |  | Bacterial invasion of epithelial cells |  | *ITGB1, PIK3CD, PIK3R3, ARHGAP10, CTNNA3* |
| 4.93E-02 |  | 0.060 |  | NF-kappa B signaling pathway |  | *BIRC2, TNFAIP3, PIDD1, TRADD, TLR4, BTK* |
| **Others** | | | | | | |
| 4.28E-14 |  | 0.063 |  | Metabolic pathways |  | *NDUFB9, CKB, GNPDA1, GPAT2, NQO1, DHTKD1, TPI1, PAH, TAT, PNPLA2, PIK3CD, INPP4B, PLA2G7, ATP6V0D2, B3GALT4, NPR2, GPI, CDO1, ATP6V1H, ATIC, PCYT1B, ADCY2, ETNPPL, PKM, UGCG, GAPDH, FAH, ACSBG1, ACSBG2, ACADM, PANK3, DGAT2, GATM, B4GALT5, PGK1, ACSF3, UAP1, PGM1, DDC, MCCC1, IVD, LCT, CHAC1, MTHFD2, DHRS9, ST8SIA1, ATP6V0A4, BTD, DTYMK, PTDSS2, GAMT, GLA, GADL1, FAHD1, DHFR, BCO1, ACSS3, NMRK2, PLCD3, ADA, CMBL, GALNT13, PSPH, TSTA3, PIP5K1B, AK7, AGPAT4, AGPAT3, AGPAT2, CCBL1, ST6GAL2, PCBD2, HYI, PCBD1, DERA, LPIN3, ASAH2, DGKH, CARNS1, AMD1, ALDH3B1, PIGL, ALDOC, ST6GALNAC3, PLA2G4F, ARSB, AKR1D1, CA4, MGAT4D, NDUFS6* |
| 1.38E-07 |  | 0.103 |  | Regulation of actin cytoskeleton |  | *ITGA8, PDGFRA, EGF, PIK3CD, GNG12, FGF19, PIK3R3, PPP1R12A, FGF16, GNA13, ITGB1, MYLK4, MYL10, MRAS, SSH2, FGFR4, FGFR1, BDKRB1, MYL12B, PIP5K1B, F2R, GIT1* |
| 3.11E-07 |  | 0.118 |  | Oxytocin signaling pathway |  | *NFATC1, NFATC2, PRKAG2, KCNJ6, CAMK1G, KCNJ12, CAMK2D, GNAO1, MYLK4, CALM1, NPR2, PIK3R6, PPP1R12A, RGS2, ADCY2, PLA2G4F, TRPM2, PRKAB1* |
| 1.90E-06 |  | 0.077 |  | Neuroactive ligand-receptor interaction |  | *GRIA1, GPR50, P2RX1, TSHR, ADCYAP1R1, CNR2, GRIN2C, RXFP3, CHRNB4, ADRA2B, PTGER3, PENK, LEPR, NPY2R, CHRNA3, GABRB2, ADM, TACR2, BDKRB1, GPR156, NTS, F2RL1, F2R, SSTR4, CHRND, HTR1F* |
| 2.85E-06 |  | 0.099 |  | Axon guidance |  | *SEMA3A, ITGB1, NFATC2, PLXNB1, MYL12B, EPHA3, ABLIM1, CAMK2D, EPHA6, PIK3CD, WNT4, SSH2, PIK3R3, TRPC5, TRPC6, NTNG2, SEMA4D, NTN1* |
| 1.53E-05 |  | 0.096 |  | cGMP-PKG signaling pathway |  | *NFATC1, NFATC2, NPR2, ADCY2, CALM1, MYLK4, KCNJ8, GTF2IRD1, ADRA2B, PIK3R6, TRPC6, PPP1R12A, RGS2, ATF4, ATP1B4, GNA13* |
| 2.29E-05 |  | 0.088 |  | Calcium signaling pathway |  | *BDKRB1, TRDN, PDGFRA, ADCY2, CAMK1G, F2R, CAMK2D, MYLK4, CALM1, ASPH, CASQ2, PTGER3, GRIN2C, CACNA1H, PLCD3, P2RX1, TACR2* |
| 6.52E-05 |  | 0.107 |  | Cholinergic synapse |  | *KCNJ12, KCNJ6, ADCY2, CAMK2D, GNAO1, PIK3CD, CHRNB4, GNG12, PIK3R6, PIK3R3, CHRNA3, ATF4* |
| 2.33E-04 |  | 0.075 |  | cAMP signaling pathway |  | *NFATC1, ADCY2, F2R, CAMK2D, ATP1B4, GRIA1, PIK3CD, PTGER3, RAPGEF4, GRIN2C, PIK3R3, PPP1R12A, TSHR, HTR1F, ADCYAP1R1, CALM1* |
| 2.70E-04 |  | 0.091 |  | Vascular smooth muscle contraction |  | *ACTA2, PRKCH, NPR2, ADCY2, MYH11, MYLK4, CALM1, RAMP3, PPP1R12A, ADM, PLA2G4F, GNA13* |
| 3.46E-04 |  | 0.103 |  | Circadian entrainment |  | *CACNA1H, KCNJ6, ADCY2, CALM1, CAMK2D, GNAO1, GRIA1, GNG12, GRIN2C, ADCYAP1R1* |
| 3.70E-04 |  | 0.088 |  | Apelin signaling pathway |  | *ACTA2, PRKAG2, GABARAPL1, ADCY2, CALM1, MYLK4, PRKAB1, MRAS, GNG12, PIK3R6, CTGF, GNA13* |
| 4.20E-04 |  | 0.081 |  | Cellular senescence |  | *NFATC1, NFATC2, CALM1, CCNE2, RAD9B, PIK3CD, MRAS, HIPK2, PIK3R3, MAP2K6, FOXM1, CCNB1, CHEK1* |
| 1.08E-03 |  | 0.107 |  | Biosynthesis of amino acids |  | *TAT, PSPH, GAPDH, TPI1, PAH, ALDOC, PGK1, PKM* |
| 1.08E-03 |  | 0.107 |  | Gastric acid secretion |  | *SLC9A4, KCNJ10, ADCY2, CALM1, CAMK2D, MYLK4, KCNK2, ATP1B4* |
| 1.62E-03 |  | 0.235 |  | Phenylalanine metabolism |  | *PAH, TAT, ALDH3B1, DDC* |
| 1.63E-03 |  | 0.113 |  | Longevity regulating pathway-multiple species |  | *HSPA2, PRKAG2, ADCY2, PRKAB1, HSPA8, PIK3CD, PIK3R3* |
| 2.72E-03 |  | 0.200 |  | One carbon pool by folate |  | *DHFR, MTFMT, ATIC, MTHFD2* |
| 2.87E-03 |  | 0.076 |  | Dopaminergic synapse |  | *PPP2R3A, KCNJ6, CALM1, CAMK2D, GNAO1, GRIA1, GNG12, PPP2R2B, DDC, ATF4* |
| 2.93E-03 |  | 0.090 |  | GABAergic synapse |  | *GABARAPL1, KCNJ6, ADCY2, SLC38A2, GNAO1, GNG12, SLC12A5, GABRB2* |
| 3.13E-03 |  | 0.089 |  | Protein digestion and absorption |  | *KCNJ13, COL10A1, SLC38A2, ATP1B4, COL14A1, COL21A1, COL12A1, COL17A1* |
| 3.14E-03 |  | 0.064 |  | Proteoglycans in cancer |  | *ITGB1, TIMP3, CAMK2D, PIK3CD, MRAS, WNT4, GPC3, PIK3R3, PPP1R12A, TLR4, WNT6, COL21A1, FGFR1* |
| 3.36E-03 |  | 0.139 |  | Tyrosine metabolism |  | *TAT, FAHD1, ALDH3B1, DDC, FAH* |
| 3.71E-03 |  | 0.079 |  | Glutamatergic synapse |  | *ADCY2, SLC38A2, GNAO1, GRIA1, GNG12, GRIN2C, HOMER3, PLA2G4F, ADRBK2* |
| 4.36E-03 |  | 0.093 |  | Glioma |  | *PDGFRA, CAMK1G, CALM1, CAMK2D, PIK3CD, PIK3R3, EGF* |
| 4.77E-03 |  | 0.082 |  | Prostate cancer |  | *PDGFRA, CCNE2, PIK3CD, PIK3R3, ATF4, LEF1, EGF, FGFR1* |
| 6.23E-03 |  | 0.154 |  | Folate biosynthesis |  | *PCBD2, DHFR, PCBD1, PAH* |
| 7.46E-03 |  | 0.070 |  | Osteoclast differentiation |  | *NFATC1, NFATC2, SPI1, SOCS3, PIK3CD, FHL2, PIK3R3, MAP2K6, BTK* |
| 9.67E-03 |  | 0.090 |  | Long-term potentiation |  | *RPS6KA3, CALM1, CAMK2D, GRIA1, GRIN2C, ATF4* |
| 1.03E-02 |  | 0.088 |  | Amphetamine addiction |  | *CALM1, CAMK2D, GRIA1, GRIN2C, DDC, ATF4* |
| 1.07E-02 |  | 0.078 |  | Hypertrophic cardiomyopathy (HCM) |  | *ITGA8, ITGB1, PRKAG2, PRKAB1, TPM4, TPM2, SGCB* |
| 1.19E-02 |  | 0.400 |  | Phenylalanine, tyrosine and tryptophan biosynthesis |  | *PAH, TAT* |
| 1.25E-02 |  | 0.075 |  | GnRH signaling pathway |  | *ADCY2, CALM1, CAMK2D, MAP3K4, MAP2K6, ATF4, PLA2G4F* |
| 1.28E-02 |  | 0.053 |  | Endocytosis |  | *HSPA2, PDGFRA, HSPA8, EPS15, PIP5K1B, WWP1, ADRBK2, UBB, IQSEC1, IQSEC3, FGFR4, SH3GL3, GIT1* |
| 1.43E-02 |  | 0.067 |  | Neurotrophin signaling pathway |  | *RPS6KA3, CALM1, CAMK2D, PIK3CD, ARHGDIG, PIK3R3, CCK, ATF4* |
| 1.74E-02 |  | 0.078 |  | Arrhythmogenic right ventricular cardiomyopathy (ARVC) |  | *ITGA8, ACTN2, SGCB, ITGB1, LEF1, CTNNA3* |
| 2.74E-02 |  | 0.070 |  | Cardiac muscle contraction |  | *TRDN, CASQ2, ATP1B4, TPM4, ASPH, TPM2* |
| 2.90E-02 |  | 0.058 |  | Ubiquitin mediated proteolysis |  | *BRCA1, KLHL13, SOCS3, WWP1, TRIM37, BIRC2, PARK2, RHOBTB2* |
| 3.14E-02 |  | 0.067 |  | Longevity regulating pathway |  | *ADCY2, PRKAB1, PRKAG2, PIK3CD, PIK3R3, ATF4* |
| 3.31E-02 |  | 0.061 |  | Serotonergic synapse |  | *KCNJ6, GNAO1, GNG12, PLA2G4F, DDC, GABRB2, HTR1F* |
| 3.43E-02 |  | 0.066 |  | Morphine addiction |  | *KCNJ6, ADCY2, GNAO1, GNG12, GABRB2, ADRBK2* |
| 3.57E-02 |  | 0.060 |  | Carbon metabolism |  | *GPI, PSPH, GAPDH, TPI1, ALDOC, PGK1, PKM* |
| 3.72E-02 |  | 0.072 |  | Central carbon metabolism in cancer |  | *PIK3CD, PDGFRA, PKM, PIK3R3, FGFR1* |
| 3.93E-02 |  | 0.050 |  | Viral carcinogenesis |  | *CCNE2, PIK3CD, TRADD, ATF4, PIK3R3, ATP6V0D2, HDAC10, CHEK1, VAC14, PKM* |
| 4.02E-02 |  | 0.083 |  | Valine, leucine and isoleucine degradation |  | *IVD, ACSF3, ACADM, MCCC1* |
| 4.19E-02 |  | 0.054 |  | Retrograde endocannabinoid signaling |  | *KCNJ6, NDUFB9, GNAO1, GRIA1, GNG12, NDUFS6, GABRB2, ADCY2* |
| 4.22E-02 |  | 0.063 |  | Dilated cardiomyopathy (DCM) |  | *ITGA8, ITGB1, ADCY2, TPM4, TPM2, SGCB* |
| 4.27E-02 |  | 0.082 |  | Cysteine and methionine metabolism |  | *TAT, CCBL1, CDO1, AMD1* |
| 4.50E-02 |  | 0.068 |  | Platinum drug resistance |  | *BRCA1, PIK3CD, XPA, PIK3R3, BIRC2* |
| 4.52E-02 |  | 0.080 |  | Arginine and proline metabolism |  | *GATM, GAMT, CARNS1, AMD1* |

^1^ Abbreviations: W, weeks of age.

^2^ Abbreviations: DEGs, different expression genes.
